# DNA methylation analysis explores the molecular basis of plasma cell-free DNA fragmentation

**DOI:** 10.1038/s41467-023-35959-6

**Published:** 2023-01-18

**Authors:** Yunyun An, Xin Zhao, Ziteng Zhang, Zhaohua Xia, Mengqi Yang, Li Ma, Yu Zhao, Gang Xu, Shunda Du, Xiang’an Wu, Shuowen Zhang, Xin Hong, Xin Jin, Kun Sun

**Affiliations:** 1 Institute of Cancer Research, Shenzhen Bay Laboratory, 518132 Shenzhen, China; 2 Hepato-Biliary Surgery Division, Shenzhen Third People’s Hospital, The Second Affiliated Hospital, Southern University of Science and Technology, 518100 Shenzhen, China; 3 Thoracic Surgical Department, Shenzhen Third People’s Hospital, The Second Affiliated Hospital, Southern University of Science and Technology, 518100 Shenzhen, China; 4 Molecular Cancer Research Center, School of Medicine, Shenzhen Campus of Sun Yat-sen University, Sun Yat-sen University, 518107 Shenzhen, China; 5 Department of Liver Surgery and Liver Transplant Center, West China Hospital of Sichuan University, 610041 Chengdu, China; 6 Department of Liver Surgery, Peking Union Medical College Hospital, PUMC and Chinese Academy of Medical Sciences, 100730 Beijing, Dongcheng China; 7 Department of Biochemistry, School of Medicine, Southern University of Science and Technology, 518055 Shenzhen, China; 8 BGI-Shenzhen, 518083 Shenzhen, China; 9 School of Medicine, South China University of Technology, 510006 Guangzhou, Guangdong China

**Keywords:** Cancer genetics, Diagnostic markers, Cancer genetics, Tumour biomarkers

## Abstract

Plasma cell-free DNA (cfDNA) are small molecules generated through a non-random fragmentation procedure. Despite commendable translational values in cancer liquid biopsy, however, the biology of cfDNA, especially the principles of cfDNA fragmentation, remains largely elusive. Through orientation-aware analyses of cfDNA fragmentation patterns against the nucleosome structure and integration with multidimensional functional genomics data, here we report a DNA methylation – nuclease preference – cutting end – size distribution axis, demonstrating the role of DNA methylation as a functional molecular regulator of cfDNA fragmentation. Hence, low-level DNA methylation could increase nucleosome accessibility and alter the cutting activities of nucleases during DNA fragmentation, which further leads to variation in cutting sites and size distribution of cfDNA. We further develop a cfDNA ending preference-based metric for cancer diagnosis, whose performance has been validated by multiple pan-cancer datasets. Our work sheds light on the molecular basis of cfDNA fragmentation towards broader applications in cancer liquid biopsy.

## Abstract (web summary)

Cell free DNA is produced through a non-random process. Here, the authors use orientation-aware fragmentation analysis to identify DNA methylation as a regulator of nuclease function.

## Introduction

Plasma cell-free DNA (cfDNA) molecules circulating in human peripheral blood are first discovered in 1948^1^. Following this discovery, the presence of tumor-, fetus-, and donor-derived DNA molecules in cancer patients, pregnant women, and organ-transplantation recipients, respectively, has led to a new era of blood-based liquid biopsy that utilizes cfDNA to perform noninvasive cancer diagnosis, prenatal testing, as well as transplantation monitoring^2–5^. Despite the impressive success in translational medicine^5^, the molecular biology of cfDNA is much less explored. Besides natural fluctuations^6^, studies have revealed that the release of cfDNA is affected by various factors, including physical activity^7^, psychosocial and physical stress conditions^8^, as well as tissues of origin under specific physiological conditions (e.g., pregnancy and cancer)^9^; however, the principles of cfDNA generation still remain elusive.

CfDNA molecules are short fragments generated through a non-random procedure^10–13^. Hallmarks of cfDNA fragmentation patterns include a major peak at 166 bp and 10-bp periodicity below 143 bp, which characteristics had been hypothesized to correlate with the nucleosome structure^14^. Other well-studied cfDNA fragmentation features include nucleosome footprints^15^, tissue-specific preferred ends^16,17^, end motif preferences^18,19^, as well as coverage/end imbalance in regulatory elements^15,20–23^. CfDNA mainly originate from cell death^24,25^, and recent studies have uncovered various crucial nucleases evolving in DNA fragmentation, including DFFB (DNA fragmentation factor subunit beta), DNASE1 (deoxyribonuclease 1), and DNASE1L3 (deoxyribonuclease 1 like 3)^26^. Different roles and preferences of these nucleases were also reported. For example, DFFB cleaves double-strand DNA into high molecular weight fragments and then into oligo-nucleosomal fragments, DNASE1 prefers to cleave nucleosome-free naked DNA, and DNASE1L3’s activity is correlated with DNA methylation^27,28^. However, to date, the underline molecular mechanisms/regulators interacting with these nucleases are still unclear. Moreover, various fundamental questions related to cfDNA fragmentation patterns remain to be answered. For instance, fetus- and tumor-derived cfDNA molecules are both shorter than the background ones^29,30^, is this phenomenon resulted from a universal molecular mechanism?

In our previous study^31^, through analyzing the size distribution of Tn5 transposase digested DNA (via ATAC-seq experiments^32^), we found that nucleosome accessibility affects cfDNA fragment end cutting preferences^31^, and cfDNA molecules of different sizes in maternal plasma of pregnant women are preferentially cut from different positions in relative to the nucleosome structure. In another previous study^33^, we reported a correlation between cfDNA size and DNA methylation density. Considering that cfDNA molecules are fragmented by various nucleases^28^, in this work, we further explore the underline molecular bases of cfDNA fragmentation via integrating of multidimensional functional genomics data. We show that DNA methylation is a regulator of nucleases’ cutting preferences that link cfDNA size and ends, which could biomarkers for cancer liquid biopsy.

## Results

### Relationship between cfDNA size and fragment end

**Fig. 1:**
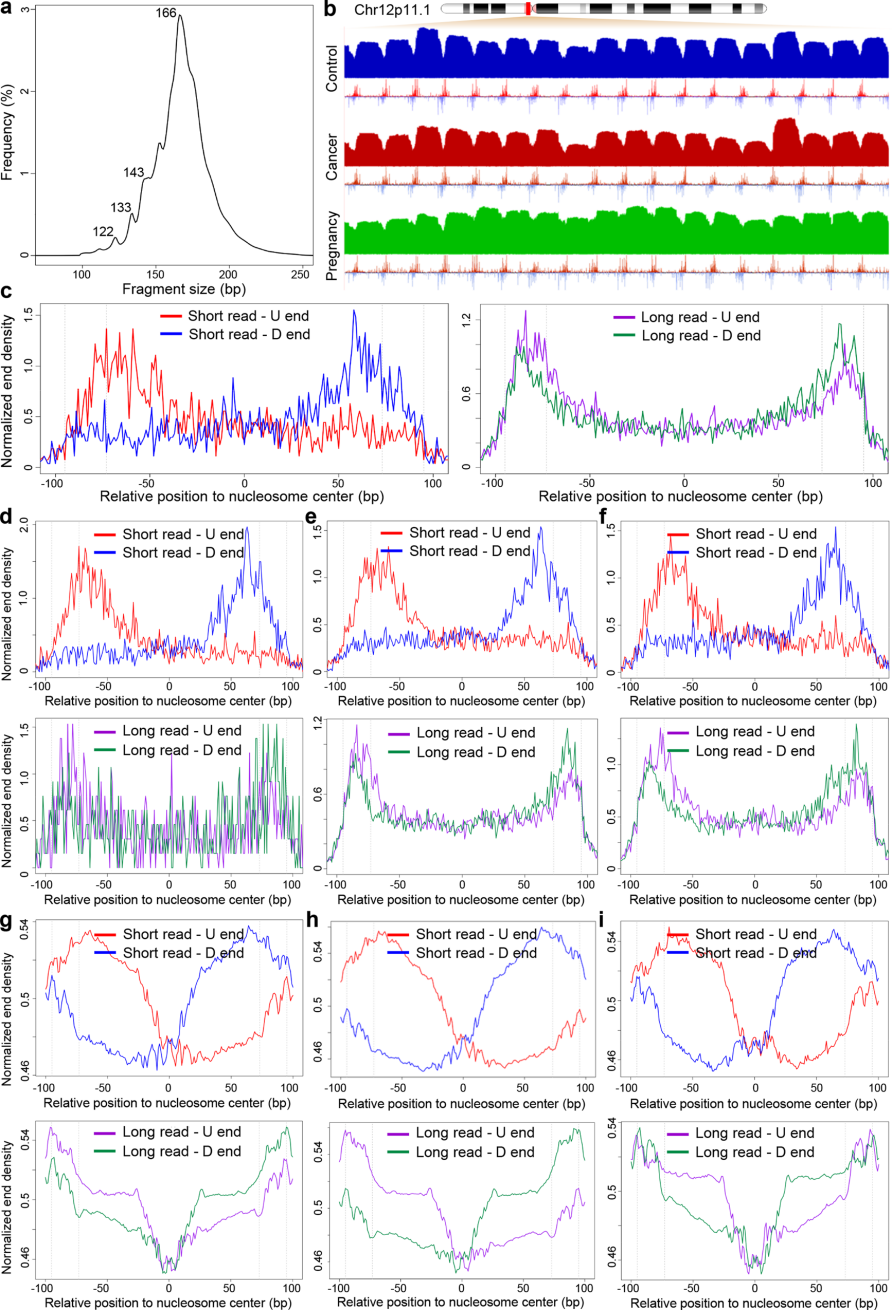
Inherent relationship between cfDNA end and size. **a** Typical cfDNA size distribution from a healthy control subject. **b** Coverage and orientation-aware fragmentation end pattern in chr12p11.1 loci. Healthy controls, breast cancer patients (from Cristiano et al. dataset), and pregnant women (from Rabinowitz et al. dataset) were illustrated. For orientation-aware fragmentation end pattern, red and blue signals stand for upstream and downstream ends, respectively. **c**–**f** Orientation-aware fragmentation end distribution for short and long cfDNA in the nucleosomal context in chr12p11.1 loci: (**c**) healthy controls, (**d**) tumor-derived cfDNA in PDX model (using primary colon tumor), (**e**) breast cancer patients, and (**f**) pregnant women. **g**–**i** Genomewide orientation-aware cfDNA fragmentation end distribution for short and long reads in the nucleosomal context: (**g**) healthy controls, (**h**) breast cancer patients, and (**i**) pregnant women. Dashed lines in (**c**–**i**) indicated the border of nucleosome core (i.e., ±73 bp from nucleosome center) and nucleosome spacing (i.e., ±90 bp from nucleosome center).

We extended our previous work on a pregnancy model to further explore the relationship between cfDNA size and fragment end in various types of samples, such as cancer. To do this, we sequenced plasma cfDNA samples from healthy controls (*n* = 24) and colorectal carcinoma (CRC) patient-derived xenograft (PDX) mouse models (where the human-originated cfDNA molecules are purely tumor-derived; *n* = 2); in addition, we collected 5 comprehensive whole genome cfDNA sequencing datasets from the literature. Hence, Snyder et al. dataset^15^ contains controls (*n* = 2) and pancreatic cancer (*n* = 4) samples; Song et al. dataset^34^ contains controls (*n* = 7) and cancer samples from 7 cancer types (*n* = 39); Cristiano et al. dataset^35^ contains controls (*n* = 231) and cancer patients from 8 cancer types (*n* = 277); Zhang et al.^36^ and Rabinowitz et al.^37^ datasets contain pregnant samples (*n* = 1 for each dataset). Figure 1a illustrated the typical size distribution of cfDNA from a control sample in Cristiano et al. dataset^35^.

We first analyzed the chr12p11.1 loci, which region contains an array of ~400 well-positioned nucleosomes in almost all tissue types and serves as an ideal model for cfDNA fragmentation analyses^15,31,38^ (Fig. 1b). We divided the cfDNA molecules into short (i.e., ≤147 bp) and long (i.e., ≥170 bp) categories based on their size^31^; for each category, we profiled the fragment end distribution within the nucleosome structure in an orientation-aware manner (i.e., for each cfDNA molecule, its fragment ends with lower and higher values in the genome coordinates were termed as upstream (U) and downstream (D) end, respectively, and processed separately in downstream analyses)^20^. As shown in Fig. 1c–f and Supplementary Figs. S1–S3, for all kinds of samples (including healthy controls, xenografted mice, cancer patients, and pregnancies), short-size cfDNA molecules showed a significantly higher proportion of fragment ends within the nucleosome than the long-size ones (*P* < 0.0001 for both U and D ends, paired *t* test; Supplementary Fig. S4). We further extended the analysis to a genome-wide level. To do this, we annotated cfDNA fragment ends using nucleosome positioning tracks determined by micrococcal nuclease digestion with deep sequencing (MNase-seq) experiments on blood cells^38^ as hematopoietic system is a major contributor of cfDNA^9,39^. Highly consistent results to the analysis on chr12p11.1 loci were observed (Fig. 1g–i and Supplementary Figs. S1–S4), demonstrating that in all sample types investigated, cfDNA with different sizes were cut from different sites in terms of the nucleosome structure, and short-size cfDNA molecules were preferably cut within the nucleosomes.

### Relationship between fragment end and peak positions in cfDNA size profile

**Fig. 2:**
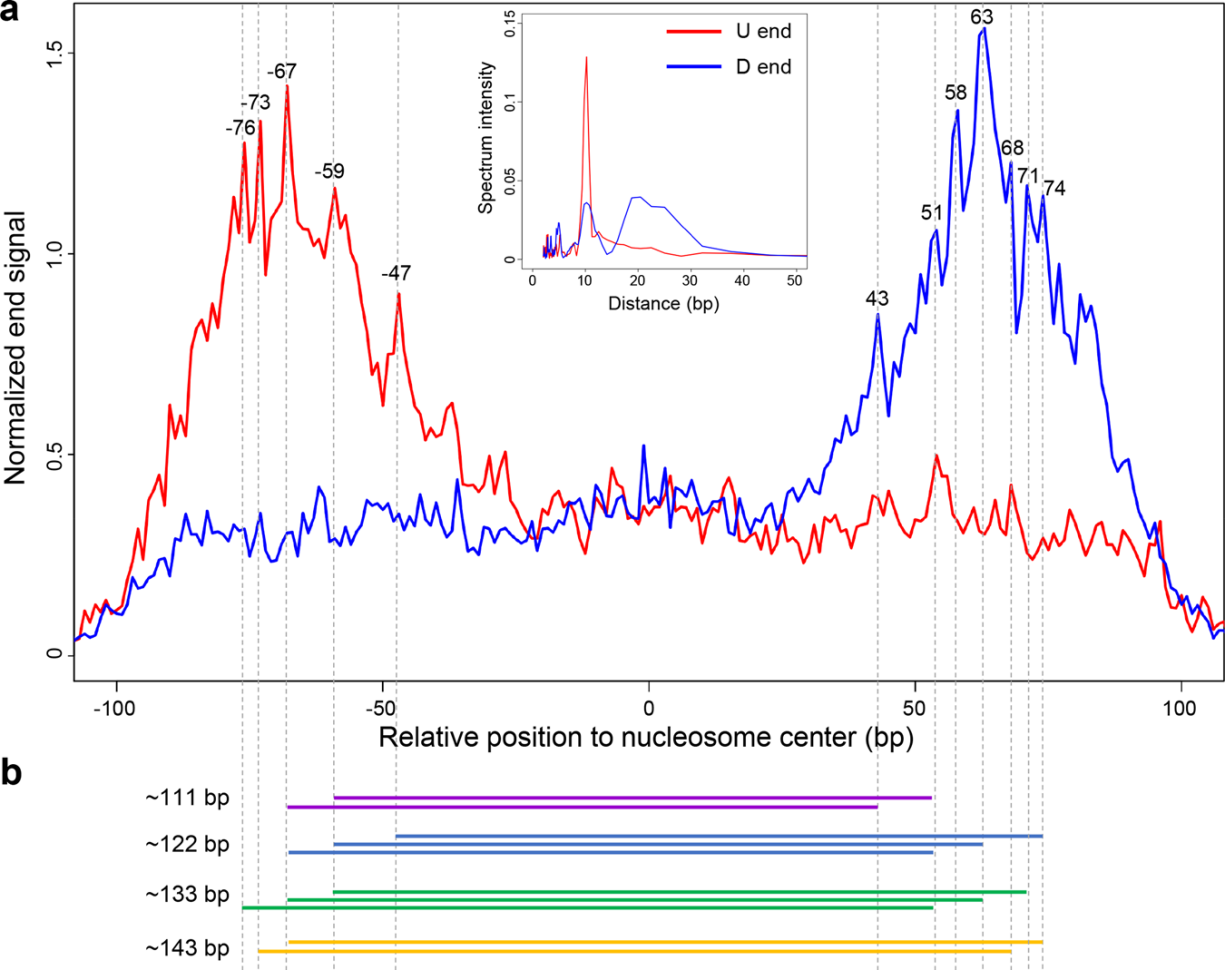
Periodicity in cfDNA fragmentation ends. **a** Orientation-aware fragmentation end distribution in the nucleosomal context in chr12p11.1 loci. Short-size cfDNA reads from pooled healthy control samples were analyzed here. Frequency analysis using FFT on the upstream (U) and downstream (D) ends were shown in the middle. **b** Combinations of the peaks in U and D ends formed the peak positions in cfDNA size characteristics.

We took a more detailed analysis on the chr12p11.1 loci, focusing on the short-size cfDNA molecules to investigate the principle of 10-bp periodicity in the size distribution of these molecules Fig. 1a). To do this, we pooled cfDNA reads from non-cancerous subjects in all datasets and reprofiled the fragment end distribution within the nucleosomal context. As a result, cfDNA fragment ends exhibited multiple peaks at certain positions (Fig. 2a): peaks for U end mostly appeared at the upper stream of nucleosome center (e.g., −68 bp), while peaks for D end mostly appeared at the downstream of nucleosome center (e.g., 63 bp), demonstrating high consistency between the U/D ends and nucleosome structure. Frequency analysis using Fast Fourier Transformation (FFT) revealed strong 10-bp periodicity in both U and D ends (Fig. 2a). As the peak positions in cutting ends corresponded to sites with higher preferences to be cut by the nucleases, we generated in silico pseudo-fragments through combinations of the peak positions in U and D ends. As a result, we found that the sizes of such pseudo-fragments coincided with the peaks in cfDNA size distribution (Fig. 2b). For instances, U end peak at −68 bp and D end peak at 74 bp would produce fragments of ~143 bp; U end peak at −59 bp and D end peak at 63 bp would produce fragments of ~122 bp. The results suggested that fragment ends might not only account for the 10-bp periodicity but also serve as determinants to the peak positions in cfDNA size characteristics.

### Relationship between DNA methylation and cfDNA fragment size

**Fig. 3:**
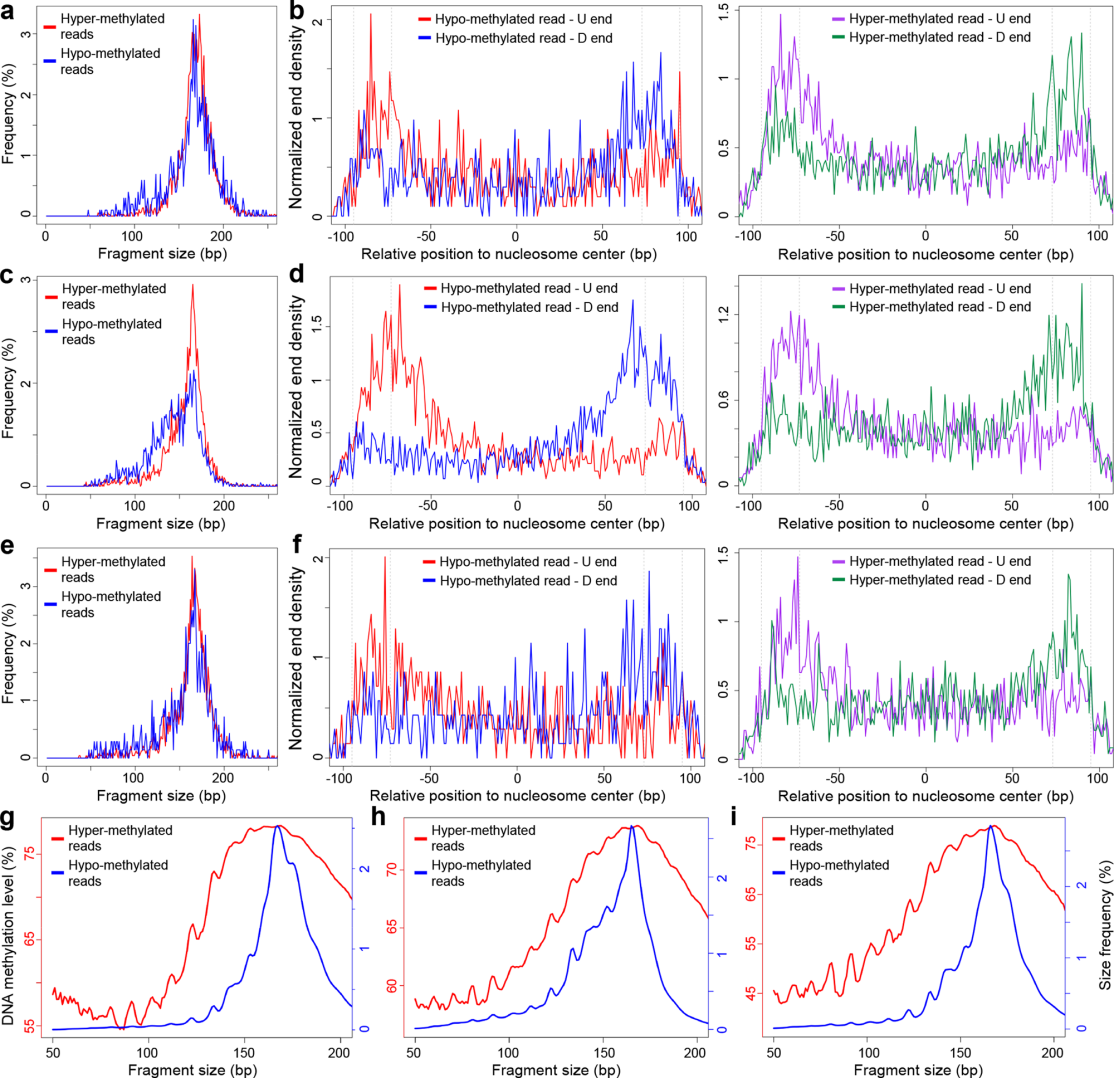
Relationship between DNA methylation and cfDNA size. **a**, **b** In control subjects, (**a**) size distribution of hyper-and hypo-methylated cfDNA reads, (**b**) orientation-aware fragmentation end distribution for hyper- and hypo-methylated cfDNA in the nucleosomal context in chr12p11.1 loci; (**c**, **d**) HCC patients, (**e**, **f**) lung adenocarcinoma patients. **g**–**i** Genomewide distribution of cfDNA size and methylation level: (**g**) control subjects, (**h**) HCC patients, (**i**) lung adenocarcinoma patients.

To explore the molecular regulator of nucleases’ preferences that determine cutting ends^31^, we performed NEBNext Enzymatic Methyl-seq (EM-seq)^40^ on plasma cfDNA from healthy controls (*n* = 5), hepatocellular carcinoma (HCC) patients (*n* = 6), and lung adenocarcinoma patients (*n* = 4). The chr12p11.1 loci were again analyzed first: we separated the cfDNA molecules into hyper-methylated and hypo-methylated categories based on the DNA methylation level of the CpG dinucleotides they carried, then profiled the cfDNA size and end distributions within the nucleosomal context for these two categories separately. The results were shown in Fig. 3a–f and Supplementary Figs. S5–S6: in both healthy controls and cancer patients, cfDNA molecules with hypo-methylated CpG dinucleotides were significantly shorter in size (*P* < 0.0001, paired *t* test). The shortness of hypo-methylated reads was validated in a public whole genome bisulfite-sequencing (WGBS) dataset generated by Zhang et al.^41^, which was composed of cfDNA samples from non-cancerous control subjects (*n* = 37), HCC patients before (*n* = 8) and after surgery (*n* = 9; *P* = 0.036, paired *t* test; Supplementary Fig. S5). Moreover, the fraction of D ends of hypo-methylated cfDNA reads within the nucleosome center was significantly increased compared to those with hyper-methylated CpG dinucleotides (*P* = 0.047, paired *t* test; Supplementary Fig. S5). In addition, we mined CpG sites that are hyper-methylated in the liver tissue while hypo-methylated in the blood cells, then investigated the size distributions of cfDNA fragments covering these CpG sites in the HCC patients. As a result, cfDNA molecules covering hypo-methylated CpG dinucleotides (mostly hematopoietic system-derived) showed significantly elevated fraction of short fragments than those with hyper-methylated CpGs (contained tumor-derived cfDNA; *P* = 0.013, paired *t* test; Supplementary Fig. S7).

We further explored the relationship of DNA methylation and cfDNA fragment size in a genome-wide manner. As shown in Fig. 3g–i and Supplementary Fig. S5, positive correlations between cfDNA fragment size and DNA methylation level were observed in both ours and Zhang et al. datasets (all *P* < 0.05, linear regression); in the meantime, DNA methylation also presented a strong 10-bp periodicity pattern with peak positions close to the size distribution, which echoed the periodicity pattern of DNA methylation pattern around the nucleosome^42^.

### Relationship between DNA methylation and nucleosome accessibility

**Fig. 4:**
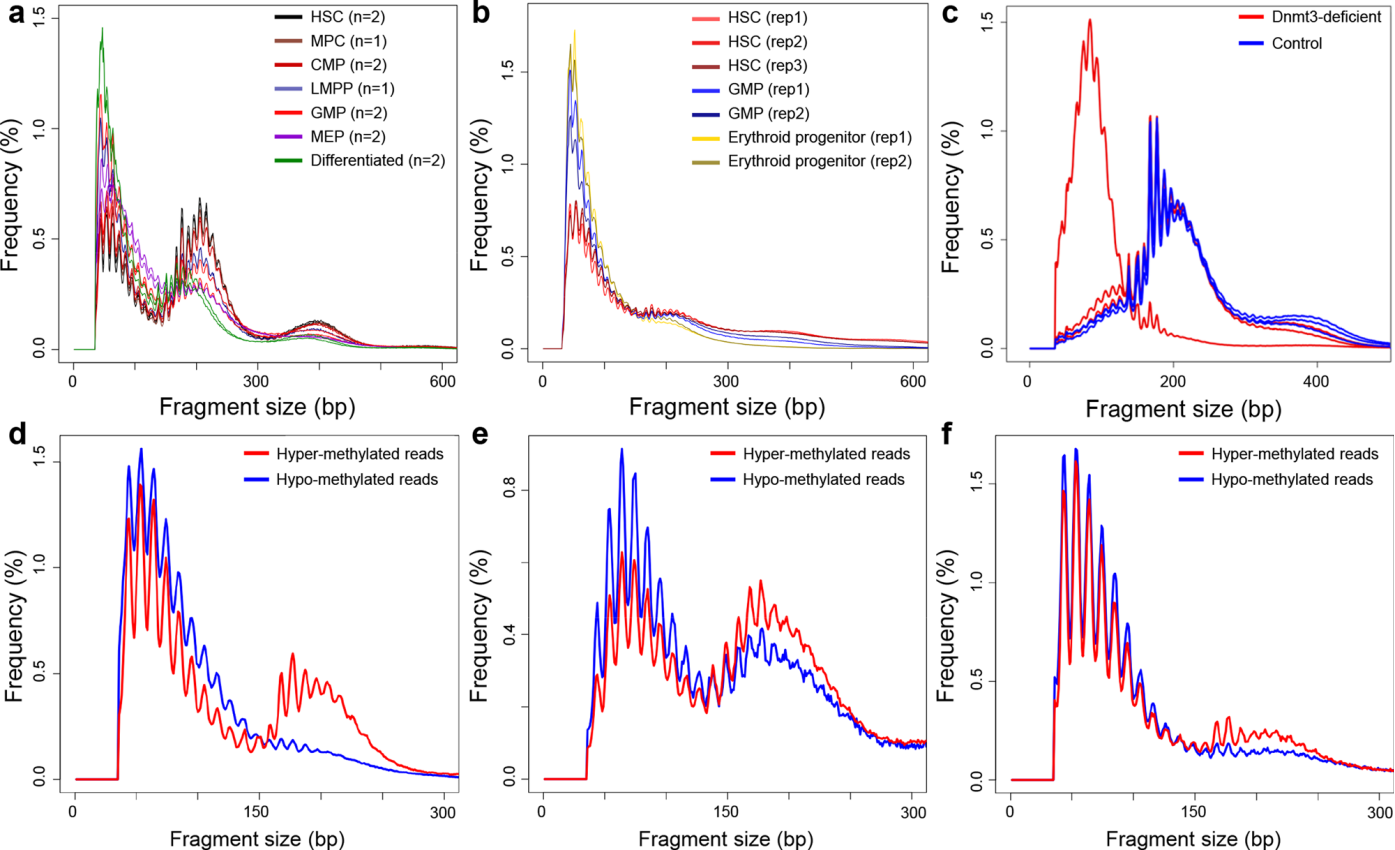
Correlation between DNA methylation and nucleosome accessibility. **a**, **b** Size distributions of Tn5-digested DNA during hematopoiesis in (**a**) Corces et al. and (**b**) Viny et al. datasets. **c** Size distribution of Tn5-digested DNA in bone marrow plasma cells in Barwick et al. Dnmt3-deficient mice model. **d**–**f** Size distributions of Tn5-digested DNA in (**d**) Barnett et al., (**e**) Izzo et al., and (**f**) Lhoumaud et al.. In (**a**) and (**b**), data from one single donor with fruitful cell types available were shown here; in (**d**–**f**), Tn5-digested DNA was divided into two groups (i.e., hyper- and hypo-methylated) based on the DNA methylation level. HSC hematopoietic stem cell, MPC multipotent progenitor cell, CMP common myeloid progenitor, LMPP lymphoid-primed multipotent progenitor cell, GMP granulocyte-macrophage progenitor, MEP megakaryocyte erythroid progenitor.

We further explored whether nucleosome accessibility is the medium linking DNA methylation and cfDNA fragmentation. Previous studies had shown that DNA methylation shows the highest level in hematopoietic stem cells (HSCs) and gradually decreases upon hematopoietic differentiation^43,44^. As shown in Fig. 4a, b and Supplementary Figs. S8, S9, hematopoietic stem cells and progenitor cells showed significantly longer fragment sizes than differentiated cells in two independent ATAC-seq datasets^45,46^ (all *P* < 0.01, *t* test). In another dataset, Barwick et al.^47^ generated a mouse model that conditionally knocks out Dnmt3a and Dnmt3b genes (i.e., Dnmt3-deficient), which encode an essential enzyme for de novo DNA methylation^48^, in B-cells. As shown in Fig. 4c and Supplementary Fig. S8, in bone marrow plasma cells where DNA methylation level was significantly decreased in Dnmt3-deficient mice, the Tn5-digested fragments was altered in Dnmt3-deficient mice compared to controls, and the peak at ~200 bp (i.e., fragments containing intact nucleosomes^32^) even disappeared in 1 Dnmt3-deficient sample (such size distribution was very similar to placental cells^31^).

To enhance the findings, we analyzed three additional datasets generated through emerging protocols that perform Tn5 digestion followed by bisulfite sequencing, which allows one to directly measure the DNA methylation level of Tn5-digested DNA. Hence, Barnett et al.^49^ and Izzo et al.^50^ performed experiments on human monocytes and hematopoietic stem cells, respectively; and the sizes of lowly methylated DNA fragments were indeed significantly shorter than highly methylated ones (*P* < 0.01 for all datasets, paired *t* test; Fig. 4d, e and Supplementary Fig. S10). Of note, in monocytes, the peak at 200 bp was almost completely absent in the low methylation DNA molecules. A similar pattern in DNA size distributions was observed in the 3rd dataset from Lhoumaud et al.^51^ working on mouse embryonic stem cells (Fig. 4f and Supplementary Figs. S10, S11).

### Alterations in end motif pattern of methylated DNA

**Fig. 5:**
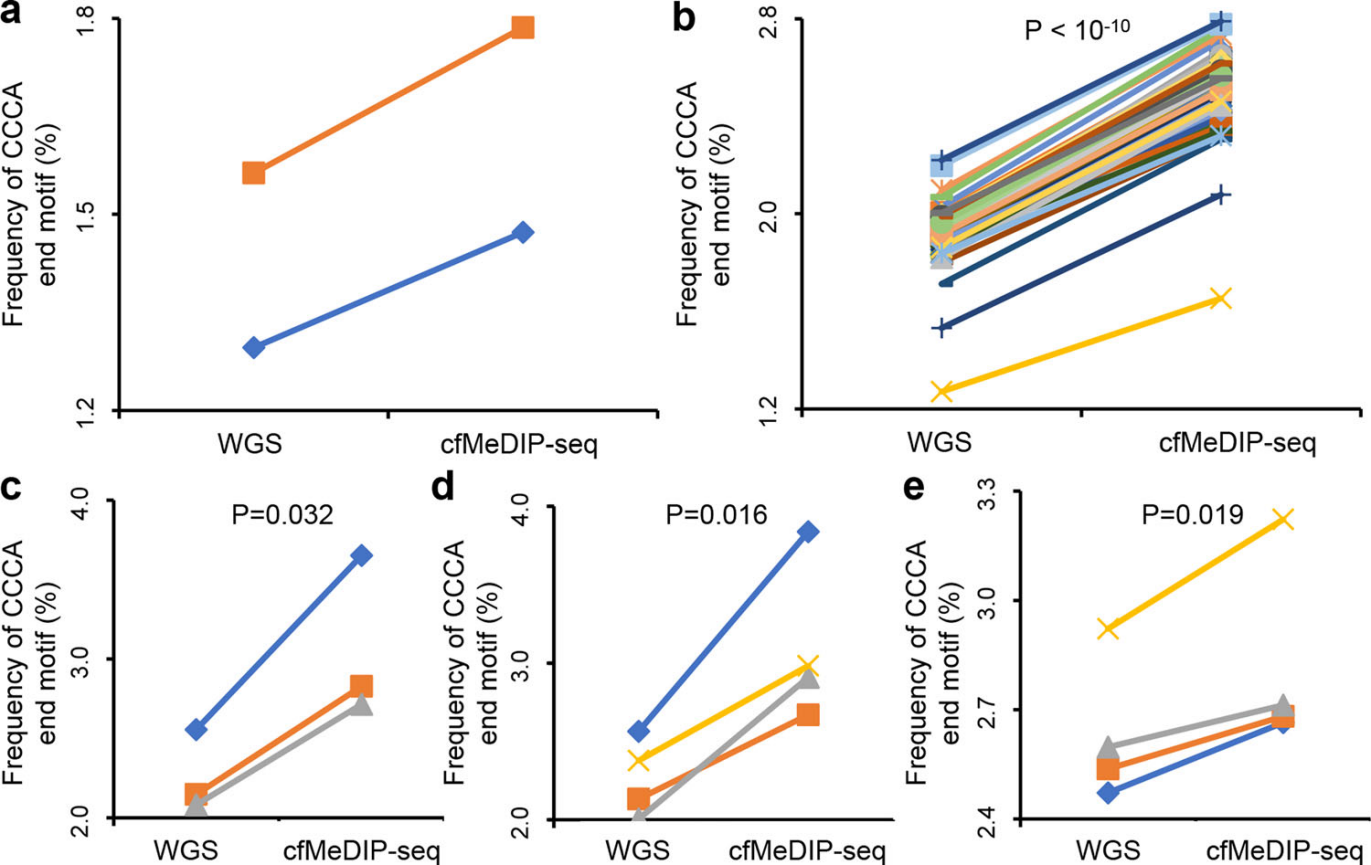
Alteration in cfDNA end motif pattern of cfMeDIP-seq data. **a** Shen et al. PDX models, **b** Peter et al. prostate cancer patients, **c** Xu et al. control subjects, **d** Xu et al. Lung cancer patients, **e** Li et al. pancreatic cancer patients. The CCCA end motif usage for paired whole genome shotgun sequencing (WGS) and cfMeDIP-seq data for all patients were shown. WGS and cfMeDIP data from the same patient was linked by a colored line. *P*-values were calculated using paired *t* test and all were two-tailed.

To further elucidate the link between DNA methylation and enzymatic cutting during apoptosis, cell-free methylated DNA immunoprecipitation-sequencing (cfMeDIP-seq)^52^ data was investigated. The cfMeDIP-seq assay captures cfDNA molecules containing methylated CpGs and therefore could enrich methylated cfDNA compared to whole genome shotgun sequencing. Three datasets with paired cfMeDIP-seq and common cfDNA shotgun sequencing data were collected. Most of the data was generated in single-end mode, which makes the analysis of cfDNA size infeasible; we therefore changed to analyze the cfDNA end motif pattern as a surrogate of nuclease cutting^19,26,27^. Hence, Shen et al.^52^ and Peter et al.^53^ datasets contain samples from xenograft mice (*n* = 2) and prostate cancer patients (*n* = 16), respectively; cfDNA end motif analysis showed that in all cases, the CCCA end motif usages in cfMeDIP-seq experiments were significantly elevated compared to paired shotgun sequencing data (*P* < 0.0001 for Peter et al. dataset; Fig. 5a, b). Such observation was validated in Li et al.^54^ and Xu et al.^55^ datasets containing control subjects (*n* = 3), lung cancer patients (*n* = 5), and pancreatic cancer patients (*n* = 4; *P* < 0.05 for all categories, paired *t* test; Fig. 5c–e).

### The E-index metric for cancer diagnosis

Considering the inherent link between cfDNA cutting end and fragment size (Figs. 1, 2), we wonder whether cfDNA end pattern could replicate the success of cfDNA size as simple, yet effective and universal biomarkers for pan-cancer diagnosis^13,56^. To do this, we profiled the frequencies of each genomic locus serving as fragment ends in our 24-case healthy control cohort to model the background cfDNA ending preference; then for each sample to evaluate, we measured the consistency level of its cfDNA ends to the model (which we called “E-index”; Supplementary Fig. S12) using a weighted average approach. In cancer patients, tumor-derived cfDNA is shorter^30^, suggesting that such molecules possess altered cutting end preference than background cfDNA; hence, we hypothesized that cancer samples would show lower E-index values than non-cancerous ones.

**Fig. 6:**
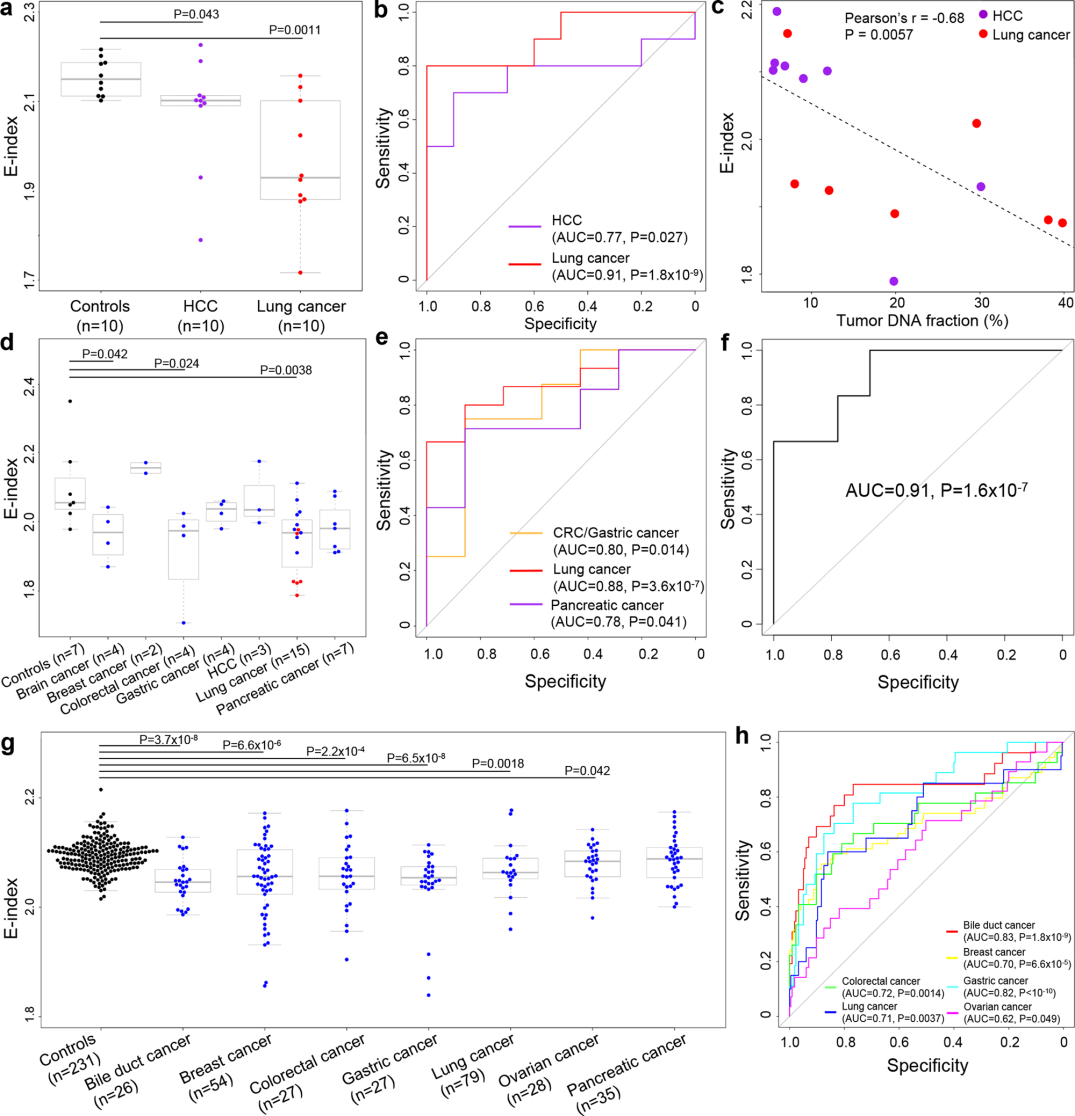
A cfDNA fragment end-based metric (E-index) for pan-cancer diagnosis. **a** E-index values in Liang et al. dataset grouped by sample types; **b** performance of E-index in cancer diagnosis on Liang et al. dataset measured using Receiver Operating Characteristics (ROC) curves; **c** correlation between E-index and tumor DNA fraction in plasma in Liang et al. dataset. **d** E-index values in Song et al. pan-cancer dataset (in lung cancer patients, red and black dots indicated patients with and without metastasis, respectively). **e** Performance of E-index in cancer diagnosis on Song et al. dataset. **f** Performance of E-index in differentiating metastatic lung cancer samples from non-metastatic ones. **g** E-index values in Cristiano et al. pan-cancer dataset (duodenal cancer was omitted from the analysis as there was only 1 sample in this category). **h** Performance of E-index in cancer diagnosis on Cristiano et al. dataset. In (**a**, **d**, **g**), *P*-values were calculated using Mann–Whitney *U* tests; center line, median; box limits, 25th and 75th percentiles; whiskers, minimum to maximum; in (**b**, **e**, **f**, **h**), *P*-values were calculated using *Z*-tests; in (**c**), *P*-value was calculated using linear regression. All *P*-values were two-sided.

To explore this hypothesis, we first investigated a previous cfDNA dataset (termed as Liang et al. dataset hereafter)^57^ generated using a similar protocol and sequencing platform as our healthy control cohort; this dataset contains healthy controls, HCC, and lung cancer patients (10 cases for each category), and E-index values for these three categories were shown in Fig. 6a. As expected, the cancer patients did show significantly decreased E-index values compared to non-cancerous controls (*P* < 0.05 for both cancer samples, Mann–Whitney *U* test); Receiver Operating Characteristics (ROC) analysis showed that E-index could readily differentiate HCC and lung cancer samples from controls with AUCs of 0.77 and 0.91, respectively (*P* < 0.05 for both cancer samples, *Z*-test; Fig. 6b). Furthermore, E-index values showed a significant negative correlation with tumor DNA load in the cancer samples (Pearson’s *r* = −0.68, *P* = 0.0057, linear regression; Fig. 6c). We further analyzed 2 pan-cancer datasets from Song et al.^34^ and Cristiano et al. (the majority of cancer samples in this dataset were in early stages)^35^. Notably, both datasets were generated using drastically different protocols and platforms than our healthy control cohort. In the Song et al. dataset, E-index values were significantly lower in most cancer samples (*P* < 0.05 for brain cancer, colorectal cancer, and lung cancer samples, Mann–Whitney *U* test) and showed remarkable capability in distinguishing cancer patients from controls (*P* < 0.05 for brain cancer, colorectal cancer, and lung cancer samples, *Z*-test; Fig. 6d–h); additionally, the metastasis information was available for lung cancer patients, and E-index values for metastatic lung cancer patients were significantly lower than non-metastatic ones (*P* = 0.0076, Mann–Whitney *U* test; Fig. 6d) along with promising power in differentiating these two categories (*P* < 0.0001, *Z*-test; Fig. 6f). Similar results to the Song et al. dataset were observed in the Cristiano et al. dataset: compared to controls, E-index were significantly decreased in bile duct cancer, breast cancer, colorectal cancer, gastric cancer, lung cancer, and ovarian cancer patients (all *P* < 0.05, Mann–Whitney *U* test; Fig. 6g); and E-index also showed capability in differentiating the cancer patients from controls (all *P* < 0.05, *Z*-test; Fig. 6h).

In addition, we re-analyzed Zhang et al. WGBS dataset, focusing on the non-cancerous controls (*n* = 37) and HCC patients (*n* = 8). As expected, both DNA methylation densities and E-index values were significantly lower in HCC patients compared to controls (both *P* < 0.001, Mann–Whitney *U* test; Supplementary Fig. S13a, b) and showed capacity in cancer diagnosis (AUCs = 0.90 and 0.96 for DNA methylation and E-index, respectively; both *P* < 0.0001, *Z*-test; Supplementary Fig. S13c), while the combination of these two features showed a higher performance than using any one alone (Supplementary Fig. S13c–e). For instance, at 100% sensitivity, the specificities for DNA methylation and E-index alone were 59.5% and 89.2%, respectively, while it was 97.3% for combination of DNA methylation and E-index.

## Discussion

The shortage in biological knowledge of cfDNA fragmentation patterns has largely limited their wider and deeper applications in cancer liquid biopsy. In this study, through integrative analyses of orientation-aware cfDNA fragment ends and various types of functional genomics data, we explored the molecular mechanism of cfDNA fragmentation patterns. We found that the fragment ends for short-sized cfDNA molecules were drastically different from those with long size (Fig. 1), and fragment ends in short-sized cfDNA molecules showed a similar 10 bp periodicity to cfDNA size pattern (Fig. 2), which was consistent our previous finding on preferred end sites in short-sized cfDNA^31^, suggesting an inherent link between cutting end and cfDNA size. In addition, cfDNA molecules of different DNA methylation levels showed drastically different sizes and end distributions (Fig. 3). In ATAC-seq datasets, we found that in the hematopoietic system, differentiated cells (with lower DNA methylation) showed an increased proportion of short, nucleosome-free fragments compared to stem cells and progenitor cells (with higher DNA methylation), and this observation was validated in experiments that incorporated Tn5 digestion and bisulfite sequencing (Fig. 4). Of note, in the Dnmt3-deficient mice from the Barwick et al. datasets, DNA methylation was significantly decreased in bone marrow plasma cells, while not in naïve and germinal center B-cells, and altered Tn5-digested fragments was indeed only observed in bone marrow plasma cells while not in the other two cell types (Fig. 4 and Supplementary Fig. S8). Results from these enzymatic digestion experiments suggested that lower DNA methylation levels predict higher nucleosome accessibility and allows nucleases to cut within nucleosomes to generate shortened DNA fragments. To date, three nucleases in human have been revealed and DNASE1L3 is the only one that has been proven to correlate with DNA methylation^26,27^. Previous studies showed that DNASE1L3 activity is linked to CCCA end motif usage in cfDNA fragments^19,26^; indeed, the CCCA end motif usage was significantly increased in cfMEDIP-seq datasets which enriched methylated cfDNA molecules (Fig. 5), suggesting that methylated DNA might be preferably cut by DNASE1L3 during DNA fragmentation. Together, the data suggested that DNA methylation might serve as a key regulator of cfDNA fragmentation, via a DNA methylation—nuclease preference—cutting end—size distribution axis. As an important epigenetic regulator, DNA methylation is strictly regulated and highly conserved in normal cells (e.g., hematopoietic system)^58^, resulting in relatively stable fragmentation characteristics of background cfDNA; as contrasts, in placental tissue and malignant cells, the overall DNA methylation level is known to be decreased compared to hematopoietic system^59,60^, suggesting that low DNA methylation might serve as a universal molecular factor to the shortness of fetus- and tumor-derived cfDNA in plasma.

To enhance the reliability of our model, in each analysis, multiple datasets from different research groups were investigated with consistent results. In addition, our findings are in line with various additional studies. For instance, previously we reported that in maternal plasma, fetus-derived cfDNA molecules get longer in late gestational stage (e.g., 3rd compared to 1st trimester), which could be well explained by the fact that DNA methylation level of placental tissue increases during pregnancy^61^. In other studies, Wang et al. reported that cfDNA fragmentation profile is altered in hypo-methylated regions in breast cancer patients^62^; Teo et al. showed that cfDNA fragments in elderly people tend to be shorter^63^, which is consistent with gradually lowering of DNA methylation during aging^64^. In chemistry, previous studies had proven that DNA methylation affects nucleosome rigidity and stability^65–67^, nuclease activity^68^, as well as the accessibility of the DNA ends of the nucleosome^69^, which might be the basis by which DNA methylation could regulate nuclease preferences during apoptotic DNA fragmentation (Figs. 4, 5). Of note, the current study focuses on double-strand cfDNA, while fragmentation schemes of single-strand cfDNA molecules could be different as suggested in previous studies^15,70–72^.

Furthermore, based on our model, we have developed and validated a cfDNA fragmentation end-based metric (i.e., E-index) for pan-cancer diagnosis using plasma cfDNA, demonstrating the potential translational value of our model in cancer liquid biopsy. Previously we had utilized statistical modeling to mine tumor-specific preferred ends in cfDNA as biomarkers for diagnosis of HCC. However, the preferred ends are only validated on HCC and whether they would work for other cancer types has not been explored yet; in addition, diagnostic models based on tumor-specific preferred ends require relatively high sequencing depth as only a limited fraction of reads are informative for diagnosis (e.g., cover the preferred end loci). As contrast, the E-index metric does not rely on complex statistical modeling and could make the most of the sequencing data; more importantly, the performance of E-index metric in pan-cancer diagnosis has been validated in multiple datasets generated using various protocols and platforms (Fig. 6). In addition, E-index could also be used along with existing biomarkers. In Zhang et al. dataset, combining E-index with DNA methylation could achieve a higher diagnostic performance than using any feature alone (Supplementary Fig. S13). Of note, most (34 out of 37) of the controls in this dataset were patients with hepatitis or cirrhosis, who were at high-risk of HCC. The results demonstrated the feasibility and merit of E-index as a promising universal biomarker for pan-cancer diagnosis and suggested that explorations on the biology of cfDNA do possess translational value, such as shedding light on efficient biomarkers for cancer diagnostics. Moreover, recent studies had validated the performance of cfDNA end-based biomarkers (such as preferred ends and end motifs) in diagnosis of HCC and lung cancers in large-scale cohorts^16,73,74^; therefore large-scale validation studies would be helpful to evaluate the translational significance of the E-index metric, either used alone or in combination with other biomarkers. In addition, to enhance the power of cfDNA fragmentomic biomarkers towards sensitive and accurate diagnosis of early-stage cancers, we believe that optimization of sequencing protocols (e.g., target enrichment of well-positioned genomic loci^15,20,38^) would be a worthwhile approach for further explorations.

As summary, in this study, we showed that DNA methylation serves as a regulator of cfDNA fragmentation. Our model shed light on the biology of cfDNA towards broader and more powerful applications in cancer liquid biopsy.

## Methods

### Ethics approval and sample processing

This study had been approved by the Ethics Committee of Shenzhen Bay Laboratory, Ethics Committee of The Third People’s Hospital of Shenzhen. Participants were recruited from The Third People’s Hospital of Shenzhen and Peking Union Medical College Hospital. Written informed consents were obtained from all participants. For each subject, 10 ml peripheral blood was collected using EDTA-containing tubes, stored at 4 °C and processed within 4 h^75^. Briefly, blood was centrifuged at 1600 *g*, 4 °C for 15 min, then the plasma portion was harvested and re-recentrifuged at 16,000 *g*, 4 °C for 15 min to remove blood cells. Plasma samples were stored at −80 °C until further usage. Tumor samples (1 from primary colon tumor, 1 from liver metastasis) were collected during surgical resections; the specimens were immediately washed using physiological saline, then stored in MACS Tissue Storage Solution (Miltenyi Biotec, #130-100-008) and implanted into immunocompromised NOD/SCID gamma (NSG) mice^76^ (8-week old) within 48 h. Animal studies were conducted according to protocols approved by the Institutional Animal Care and Use Committee, Southern University of Science and Technology. Mice were housed under specific pathogen-free conditions with a 12 h light/dark cycle, at a temperature of 20–26 °C, and a relative humidity of 40–70%; mice were fed a standard mouse chow diet.

### CfDNA extraction

For WGS and EM-seq library preparation, 600 μL and 2 mL cell-free plasma was used to extract cfDNA, respectively. CfDNA was extracted with MGIEasy Circulating DNA Isolation Kit (MGI, #1000017017). 40 μL proteinase K solution and 50 μL MGIPure particle G were added to 600 μL plasma. Then 1.1 mL Lysis buffer was added to the mixture and incubated at room temperature for 15 min. After the separation on the Magnetic Separation Rack, the supernatant was discarded, and the magnetic beads were washed with 700 μL Wash Buffer 1 and 700 μL Wash Buffer 2 twice. Then cfDNA was eluted with 35 μL water and quantified by Qubit dsDNA HS Assay Kit (Invitrogen, #Q32851) in Qubit 3 Fluorometer.

### Whole genome sequencing of cfDNA

For each sample, 600 μL plasma was used to extract cfDNA and DNA library was constructed using MGI Cell-free DNA Library Prep kit (MGI, #94000018500) following the manufacturer’s instructions. Briefly, 40 μL sample was incubated with 10 μL ERAT Mix at 37 °C for 10 min and followed by 65 °C for 15 min; then DNA was ligated with sequencing adaptors at 23 °C for 20 min and purified with 40 μL Purification Beads; purified DNA was amplified for 12 cycles with PCR Mix and purified with 1× volume of magnetic beads and quantified using Qubit dsDNA HS Assay Kit (Invitrogen, #Q32851) in Qubit 3 Fluorometer (Invitrogen, #Q33216). PCR product was denatured at 95 °C for 3 min and circulated with DNA Rapid Ligase at 37 °C for 30 min. The DNA libraries were subjected to DNA nanoball (DNB) generation and sequenced on an MGISEQ-2000 (MGI) sequencer with MGISEQ-2000RS Sequencing Reagent (MGI) in paired-end 100 bp mode (read number: median 40.8 million, range 36.1–45.0 million for healthy controls; 344.5 million and 233.7 million for the 2 PDX models). Key statistics of the data were provided in Supplementary Table S1.

### Enzymatic Methyl-seq (EM-seq) of cfDNA

EM-seq is a similar approach to WGBS^77^ that allows one to study DNA methylation at base resolution^40^. For each sample, 2 mL plasma was used to extract DNA and subjected to EM-seq library preparation using NEBNext Enzymatic Methyl-seq kit (NEB, #E7120S) following the manufacturer’s instructions. Briefly, extracted cfDNA was firstly mixed with 20 pg unmethylated lambda DNA (NEB, #E7120S; used as spike-ins for quality control), then incubated with 10 μL End Prep Mix at 20 °C for 30 min and followed by 65 °C for 30 min. The DNA then was ligated with methylated adaptors at 20 °C for 15 min, purified with 110 μL magnetic beads, and eluted with 28 μL elution buffer. The purified DNA was used for methylcytosine oxidation with the 17 μL TET2 reaction mix and 5 μL Fe (II) solution and incubated at 37 °C for 1 h. The reaction was stopped by adding 1 μL of Stop Reagent and incubating at 37 °C for 30 min. The oxidated DNA was purified with 90 μL magnetic beads and eluted in 16 μL elution buffer. 4 μL Formamide (Sigma-Aldrich, #F9037-100ML) was added to denature DNA at 85 °C for 10 min and deamination was immediately carried out by adding 80 μL APOBEC reaction mix to the tube and followed by incubation at 37 °C for 3 h. The treated DNA was then purified with 100 μl magnetic beads. Indexed primers and NEBNext Q5U Master Mix (NEB, #E7120S) were added to purified DNA for 8 cycles of amplification, and each amplified library was purified with 0.9× volume of magnetic beads. EM-seq libraries were sequenced on an NovaSeq 6000 sequencer (Illumina) in paired-end 150 bp mode (read number: median 112.8 million, range 72.4–189.1 million). Key statistics of the data were provided in Supplementary Table S1.

### Sequencing data analysis

CfDNA whole genome sequencing data, ATAC-seq data and cfMeDIP-seq data were analyzed using a unified pipeline: the raw reads were firstly preprocessed using Ktrim software^78^ to remove sequencing adapter and low-quality cycles; the preprocessed reads were then mapped to reference human genome (NCBI GRCh38) for human samples, reference mouse genome (NCBI GRCm38) for normal mouse samples, or a pseudo-genome that combined reference human and mouse genomes for PDX samples, using Bowtie2 software^79^; PCR duplications (i.e., reads with identical ending positions) were identified and removed using in-house programs, and resulting reads were collected as the final clean data. Due to the limited depth for each case, in each dataset, cfDNA samples from the same cancer type or the control group were pooled together during downstream fragmentation analyses. For PDX samples, reads mapped to human genome were considered as tumor-derived and were used in the downstream analyses. For Liang et al. dataset, tumor DNA load in plasma cfDNA was estimated using ichorCNA software^80^.

EM-seq, WGBS, and Tn5-digestion followed by bisulfite-sequencing datasets were analyzed using Msuite2 software^81,82^, which included quality control, read alignment, and methylation calling. For EM-seq and WGBS datasets, sequencing reads covering at least 2 CpG sites (Fig. 3 and Supplementary Fig. S5; or at least 5 CpG sites, Supplementary Fig. S6) with an average methylation level larger than 80% or lower than 20% were considered as hyper-methylated or hypo-methylated reads^33^, respectively. For ATAC-seq and Tn5-digestion followed by bisulfite-sequencing datasets, as we were only interested in Tn5 cutting within nucleosomes, only reads outside the peak regions (i.e., open-chromatin regions that do not have nucleosomes; obtained from the corresponding studies) were used in downstream analyses.

Nucleosome track for GM12878 cell line (lymphoblastoid lineage) was downloaded from NucMap database^83^ (https://ngdc.cncb.ac.cn/nucmap; accession number: hsNuc0390101; nucleosome occupancy and center loci were determined using DANPOS software^84^). Genomic coordination of chr12p11.1 loci was obtained from Gaffney et al.^38^ (which was provided for NCBI GRCh36 reference human genome) and converted to NCBI GRCh38 reference human genome using “liftOver” program from the UCSC genome browser. Orientation-aware fragmentation analysis were performed as previously^20^. Briefly, for each cfDNA molecule, the ends with lower and higher values in the genome coordinate were termed as U and D ends, respectively; for all nucleosomes annotated in the nucleosome track, we collected the U/D ends that fell in each nucleosome and calculated the relative positions of the U/D ends to the corresponding nucleosome center, then profiled the frequencies of the relative positions as the end distribution in nucleosomal context. Note that the genomewide cfDNA end against nucleosomal context analysis was skipped for PDX models, as the tumors were collected from a colorectal cancer patient and were not from hematopoietic system. CfDNA end motif analysis was performed as previously^18,19^. Briefly, we extracted the 4-mer sequence from the 5′-end of all cfDNA reads and calculated the frequencies of each combination; frequencies of reads with CCCA end motif were extracted and analyzed in samples with paired cfDNA whole genome sequencing and cfMeDIP-seq data.

### Fast Fourier transform (FFT) analysis

FFT analysis was performed using a similar approach as Snyder et al.^15^. Briefly, the U/D distribution signals were first de-trended by subtracting the smoothened mean (calculated using loess (locally weighted regression) function implemented in R software); the “spec.pgram” function implemented in R software was then used to determine the spectral density of each frequency.

### CfDNA ending preference model and E-index

To model the cfDNA ending preference in control subjects, we pooled all cfDNA data from the 24 healthy control cohort generated in this study and extracted the U and D ends from all reads; for each locus in the genome, we counted the appearances of serving as U or D ends in the pooled cfDNA data separately, and the resulting genomewide count table was defined as the cfDNA ending preference model. As we only built 1 model and utilized it to analyze pan-cancer cfDNA samples throughout the study, there was no need to normalize the counts to the total read number of the healthy cohort.

For each evaluated cfDNA sample, we extracted the genomic coordinates of all U and D ends and summed the corresponding counts in the cfDNA ending preference model (as weights) to calculate a consistency score, which was further normalized by the read number of the corresponding sample as illustrated in the following formula (Supplementary Fig. S12):

1 $${{{{{{{\rm{E}}}}}}}}{\mbox{-}}{{{{{\rm{index}}}}}}=\frac{1}{{{N}}}{\sum }_{{{i}}}{{{M}}}_{{{{{{\rm{U}}}}}}}+{{{M}}}_{{{{{{\rm{D}}}}}}}$$

where *N* denoted the read number of the working sample, *i* denoted each sequencing read and *M*_U_, *M*_D_ denoted the counts of its ending positions serving as U and D ends in the cfDNA ending preference model, respectively.

### Statistics and reproducibility

No statistical method was used to predetermine the sample size. The experiments were not randomized. The investigators were not blinded to allocation during experiments and outcome assessment. Parametric tests (e.g., *t* test) were used if data followed normal distributions; otherwise, non-parametric tests (e.g., Mann–Whitney *U* test) would be used. For Xu et al. dataset, 1 sample was discarded due to aberrant size pattern; for Shen et al. dataset, only the data from 2 PDX models were publicly accessible, and only the reads mapped to the mouse genome were used in motif analysis as the reads mapped to the human genome were too few.

### Reporting summary

Further information on research design is available in the Nature Portfolio Reporting Summary linked to this article.

## Supplementary information

Supplementary Information

Reporting Summary

## Data Availability

The raw sequencing data generated in this study have been deposited in the Genome Sequence Archive in National Genomics Data Center, China National Center for Bioinformation / Beijing Institute of Genomics, Chinese Academy of Sciences (GSA-Human) under accession codes HRA002237, cfDNA WGS on PDX models, HRA002250, cfDNA WGS on healthy controls, and HRA002298, cfDNA EM-seq on healthy controls, HCC and LUAD patients) under controlled access due to patient consent restrictions. Applications for data access should approach Kun Sun (sunkun@szbl.ac.cn; applicants should have obtained ethics approvals from their ethic committees; timescale for access to be granted would be around 1 month and there are no restrictions on duration of access). Source data are provided with this paper. Public cfDNA whole genome sequencing datasets were downloaded from Gene Expression Omnibus (GEO) under accession numbers GSE71378, GSE124686, and GSE81314; only the data generated using double-strand cfDNA were analyzed) and FinaleDB^85^ [http://finaledb.research.cchmc.org/]. WGBS dataset for cfDNA was downloaded from Genome Sequence Archive in National Genomics Data Center (GSA) under accession number CRA001537; only the samples with patient IDs were used); WGBS datasets for human blood cells and liver tissue were downloaded from Encylopedia of DNA Elements project (ENCODE) under accession numbers ENCSR663MXB, and ENCSR108ESU. ATAC-seq datasets for various blood cell types during hematopoietic differentiation were downloaded from GEO under accession numbers: GSE74912, GSE138003; ATAC-seq dataset for Dnmt3-decifient mouse model was downloaded from GEO under accession number GSE89471. Tn5-digestion followed by bisulfite-sequencing datasets were downloaded from GEO under accession numbers GSE130096, GSE124822, and GSE129673. CfMeDIP-seq datasets were downloaded from GEO under accession numbers GSE79838 and GSE152631, and Sequence Read Archive (SRA) under accession number SRP262262. Source data are provided with this paper.

## Source data

Source Data

## Code availability

Computational programs and scripts to reproduce the results were available at github (https://github.com/hellosunking/molecular-cfDNA-fragmentomics), and Zenodo (10.5281/zenodo.7420630)^86^.
