# Supplementary material for: DNA methylation analysis explores the molecular basis of plasma cell-free DNA fragmentation
Source: Nat Commun. 2023 Jan 18;14:287. doi: 10.1038/s41467-023-35959-6 (PMC9849216; doi:10.1038/s41467-023-35959-6)
Supplement: Supplementary file 1 — Supplementary Information [file 41467_2023_35959_MOESM1_ESM.pdf]

DNA methylation analysis explores the molecular basis of plasma cell-free DNA fragmentation

An *et al.*

This file contains Supplementary Table S1 and Supplementary Figures S1-S13.

Supplementary Table S1. Key statistics of cfDNA whole genome sequencing and EM-seq data.

Category	Sample ID	Raw reads	Ktrim preprocessed	Mappable reads	Uniquely mapped reads	
Healthy controls	CTR-1	41,042,756	40,636,236	38,104,831	37,740,155	
	CTR-2	42,696,006	42,273,067	39,661,950	38,952,261	
	CTR-3	43,575,044	43,180,620	40,763,817	40,387,369	
	CTR-4	40,565,377	40,198,849	37,753,221	37,429,667	
	CTR-5	36,135,644	35,833,342	33,791,152	33,407,873	
	CTR-6	41,116,409	40,771,861	38,186,491	37,830,865	
	CTR-7	36,500,934	36,125,375	33,694,667	33,472,259	
	CTR-8	44,130,773	43,731,628	40,957,321	40,473,628	
	CTR-9	37,618,138	37,307,258	35,139,683	34,646,739	
	CTR-10	36,184,975	35,899,229	33,643,719	33,373,052	
	CTR-11	40,231,706	39,867,364	37,506,155	37,106,248	
	CTR-12	43,997,308	43,667,507	41,175,488	40,818,610	
	CTR-13	38,135,682	37,865,520	35,925,053	35,202,134	
	CTR-14	38,895,784	38,600,000	36,528,937	36,212,147	
	CTR-15	38,693,654	38,347,451	36,133,268	35,733,737	
	CTR-16	44,952,056	44,559,892	41,953,578	41,241,074	
	CTR-17	40,351,576	39,966,047	37,621,138	37,320,398	
	CTR-18	42,158,090	41,751,351	39,002,834	38,742,477	
	CTR-19	37,332,832	36,995,907	34,619,983	34,102,572	
	CTR-20	40,133,606	39,768,290	37,252,197	36,916,885	
	CTR-21	42,783,965	42,452,117	40,166,597	39,590,196	
	CTR-22	44,209,536	43,853,807	40,991,501	40,536,201	
	CTR-23	43,295,659	42,932,762	40,419,138	39,822,020	
	CTR-24	44,021,297	43,663,341	41,169,395	40,698,229	
	**Sample ID**	**Raw reads**	**Ktrim preprocessed**	**Mappable reads**	**Uniquely mapped reads**	**Tumor-origionated**
PDX models	PDX-84	344,453,886	341,429,758	294,924,350	288,080,146	171,999,951
	PDX-85	233,754,393	232,165,478	204,801,111	179,841,147	87,559,419
	**Sample ID**	**Raw reads**	**Mappable reads**	**Overall DNA methylation (%)**		
EM-seq	Control-1	82,267,665	63,285,133	69.8		
	Control-2	165,216,237	109,025,926	73.0		
	Control-3	124,692,775	84,190,243	72.6		
	Control-4	189,085,822	127,399,994	73.6		
	Control-5	72,384,208	55,220,187	71.6		
	HCC-1	152,892,203	103,686,354	66.3		
	HCC-2	114,280,351	85,703,914	70.9		
	HCC-3	156,830,016	106,095,126	69.4		

HCC-4	81,092,330	62,407,643	72.5
HCC-5	113,834,280	86,159,178	75.2
HCC-6	112,843,645	75,344,466	67.3
LUAD-1	77,679,838	58,861,878	72.2
LUAD-2	81,885,230	62,077,410	71.6
LUAD-3	92,805,874	69,916,403	72.7
LUAD-4	74,276,382	56,962,178	74.3

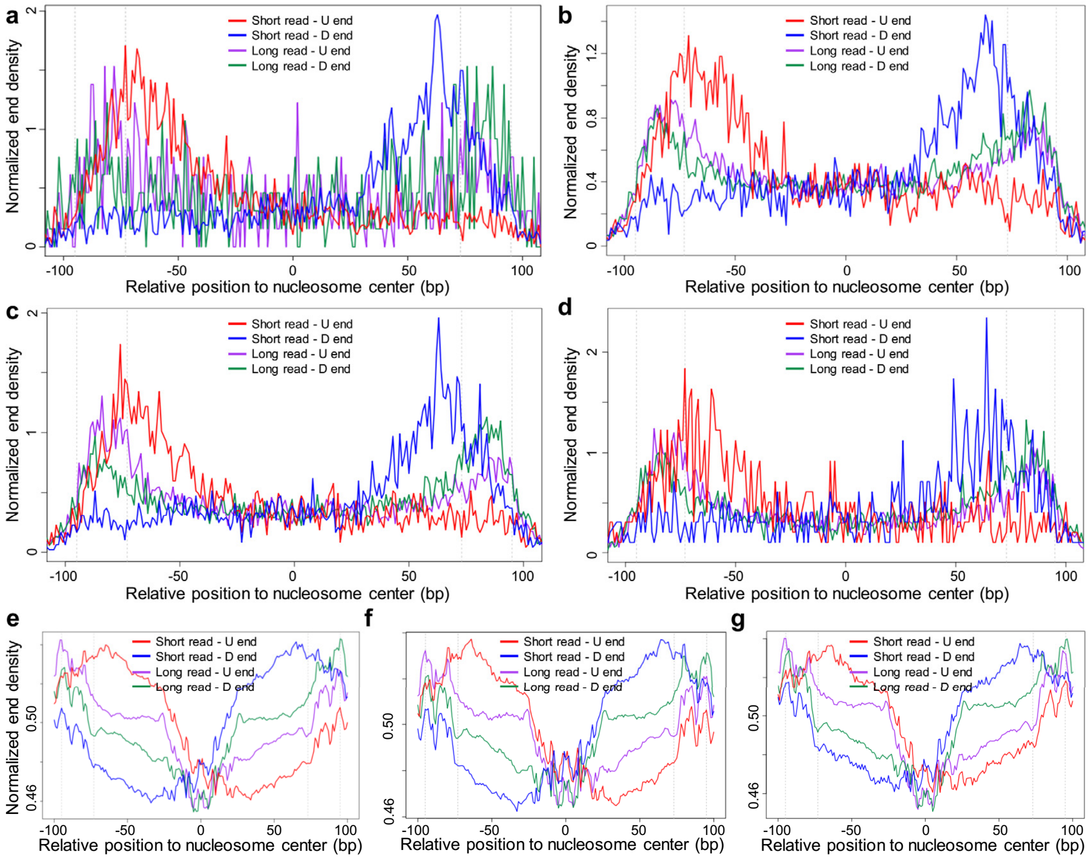

Supplementary Figure S1. Orientation-aware cfDNA end and size distribution on PDX model, Snyder *et al.* dataset and Zhang *et al.* dataset. (a-d) Orientation-aware fragmentation end distribution for short and long cfDNA in the nucleosomal context in chr12p11.1 loci: (a) PDX model using liver metastasis; (b) healthy controls from Snyder *et al.* dataset, (c) pancreatic cancer patients from Snyder *et al.* dataset, and (d) pregnant women from Zhang *et al.* dataset. (e-g) Genomewide orientation-aware cfDNA fragmentation end distribution for short and long reads in the nucleosomal context: (e) healthy controls from Snyder *et al.* dataset, (f) pancreatic cancer patients from Snyder *et al.* dataset, and (g) pregnant women from Zhang *et al.* dataset. Dashed lines indicated the border of nucleosome core (i.e., ±73 bp from nucleosome center) and nucleosome spacing (i.e., ±90 bp from nucleosome center).

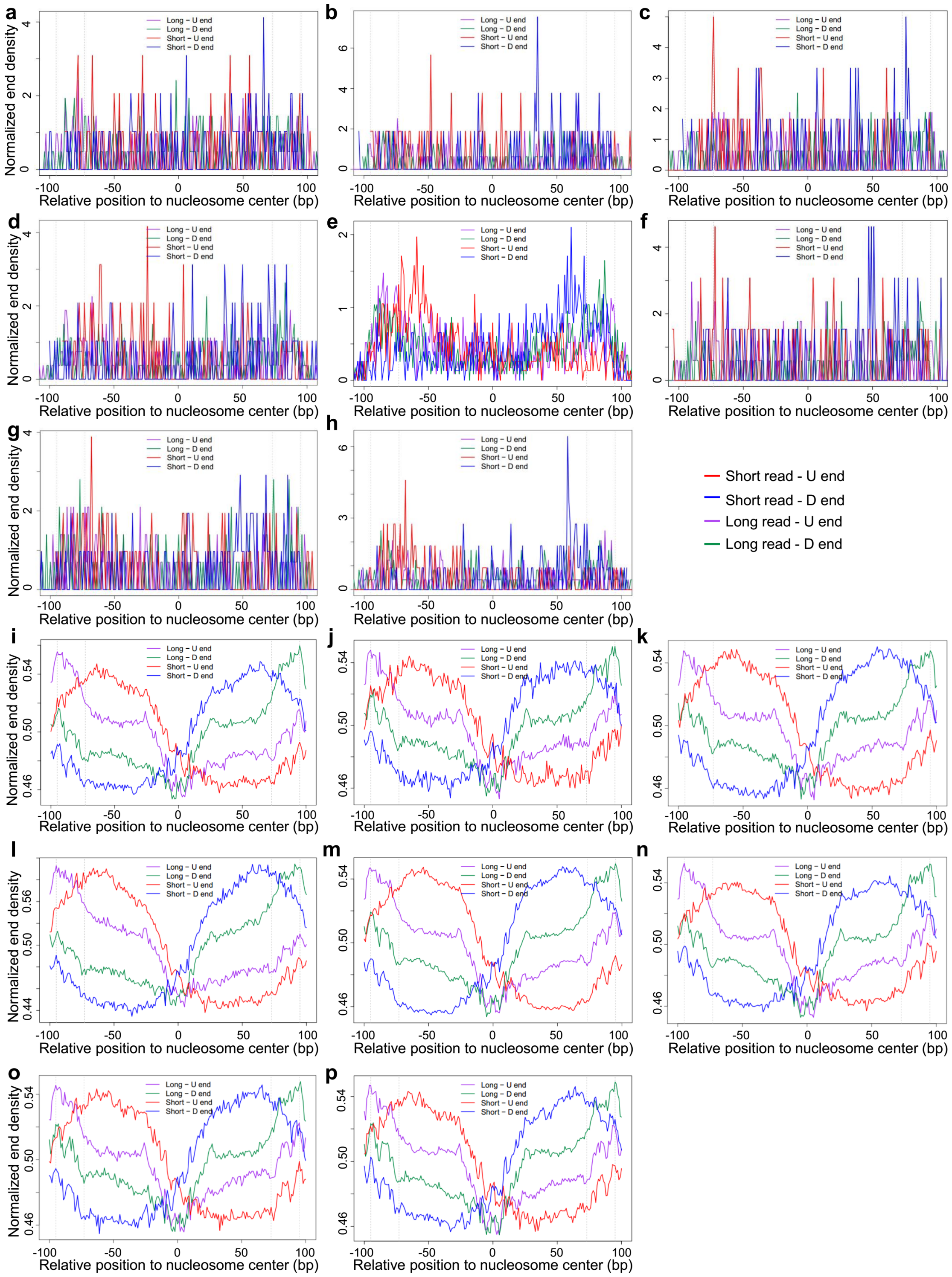

Supplementary Figure S2. Orientation-aware cfDNA end and size distribution on samples from Song *et al.* dataset. (a-h) Orientation-aware fragmentation end distribution for short and long cfDNA in the nucleosomal context in chr12p11.1 loci: (a) brain cancer, (b) breast cancer, (c) colon cancer, (d) HCC, (e) lung cancer, (f)

pancreatic cancer, (g) gastric cancer, (h) non-cancerous controls (including healthy subjects and HBV carriers). (i-p) Genomewide orientation-aware cfDNA fragmentation end distribution for short and long reads in the nucleosomal context: (i) brain cancer, (j) breast cancer, (k) colon cancer, (l) HCC, (m) lung cancer, (n) pancreatic cancer, (o) gastric cancer, (p) non-cancerous controls (including healthy subjects and HBV carriers). Dashed lines indicated the border of nucleosome core (i.e., ±73 bp from nucleosome center) and nucleosome spacing (i.e., ±90 bp from nucleosome center).

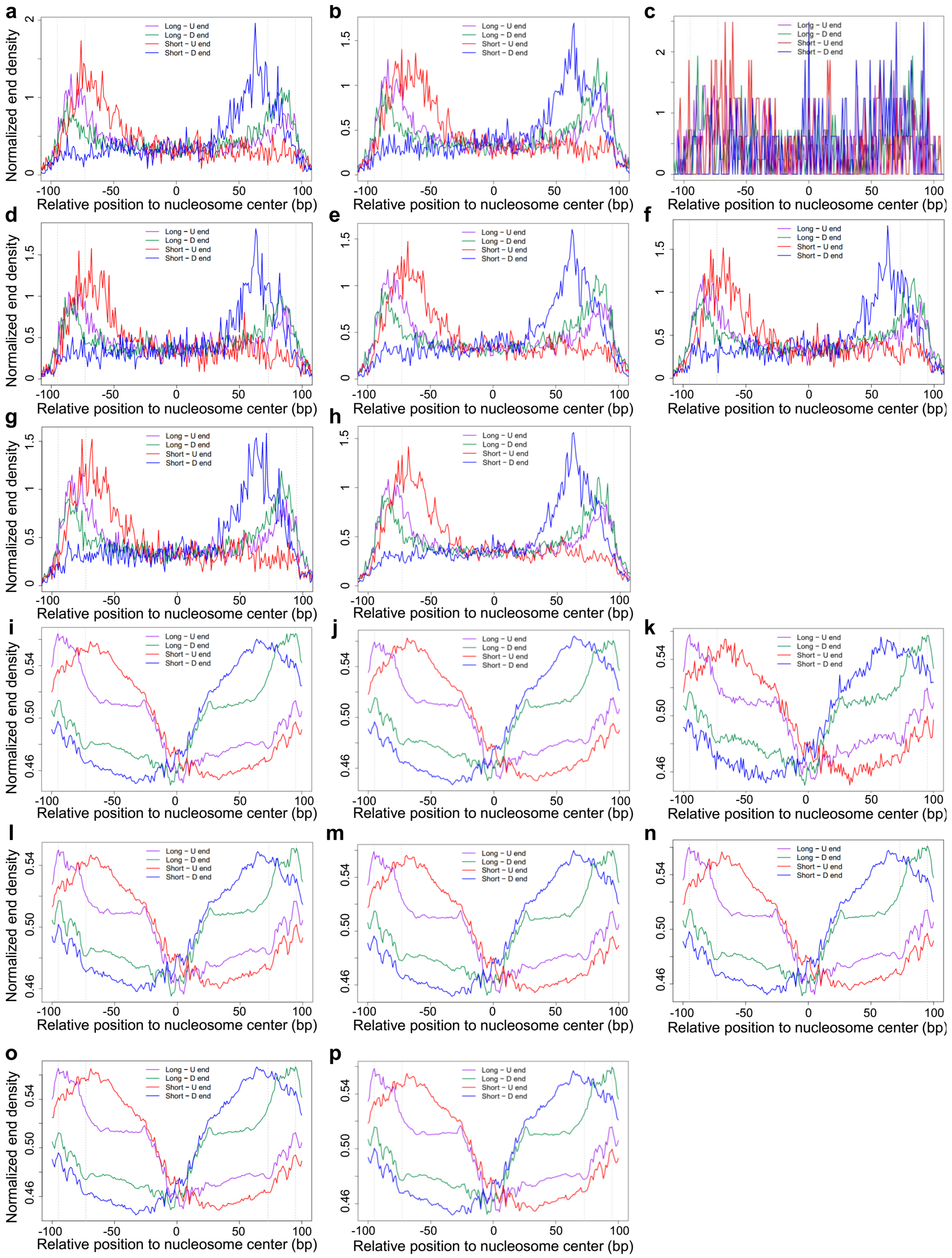

Supplementary Figure S3. Orientation-aware cfDNA end and size distribution on samples from Cristiano *et al.* dataset. (a-h) Orientation-aware fragmentation end distribution for short and long cfDNA in the nucleosomal context in chr12p11.1 loci: (a) bile duct cancer, (b) colon cancer, (c) duodenal cancer, (d) gastric cancer, (e) lung

cancer, (f) ovarian cancer, (g) pancreatic cancer, (h) non-cancerous controls. (i-p) Genomewide orientation-aware cfDNA fragmentation end distribution for short and long reads in the nucleosomal context: (i) bile duct cancer, (j) colon cancer, (k) duodenal cancer, (l) gastric cancer, (m) lung cancer, (n) ovarian cancer, (o) pancreatic cancer, (p) non-cancerous controls. Dashed lines indicated the border of nucleosome core (i.e., ±73 bp from nucleosome center) and nucleosome spacing (i.e., ±90 bp from nucleosome center). In (c), there was only 1 case with duodenal cancer, therefore the sequencing depth was much lower compared to other cancer types.

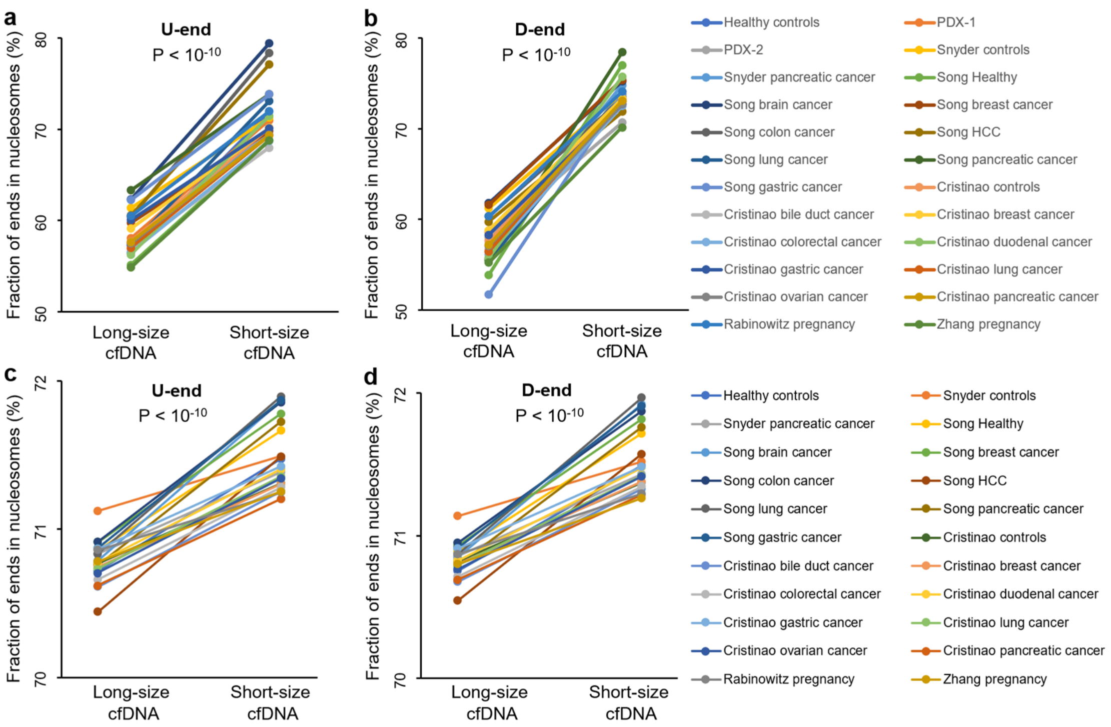

Supplementary Figure S4. Comparison of fraction of orientation-aware ends within the nucleosome in long- and short-size cfDNA. (a) U-end in chr12p11.1 (n=24 control or disease types); (b) D-end in chr12p11.1 (n=24 control or disease types); (c) U-end in whole genome (n=22 control or disease types); (d) D-end in whole genome (n=22 control or disease types). P-values were calculated using paired t-tests (two-sided).

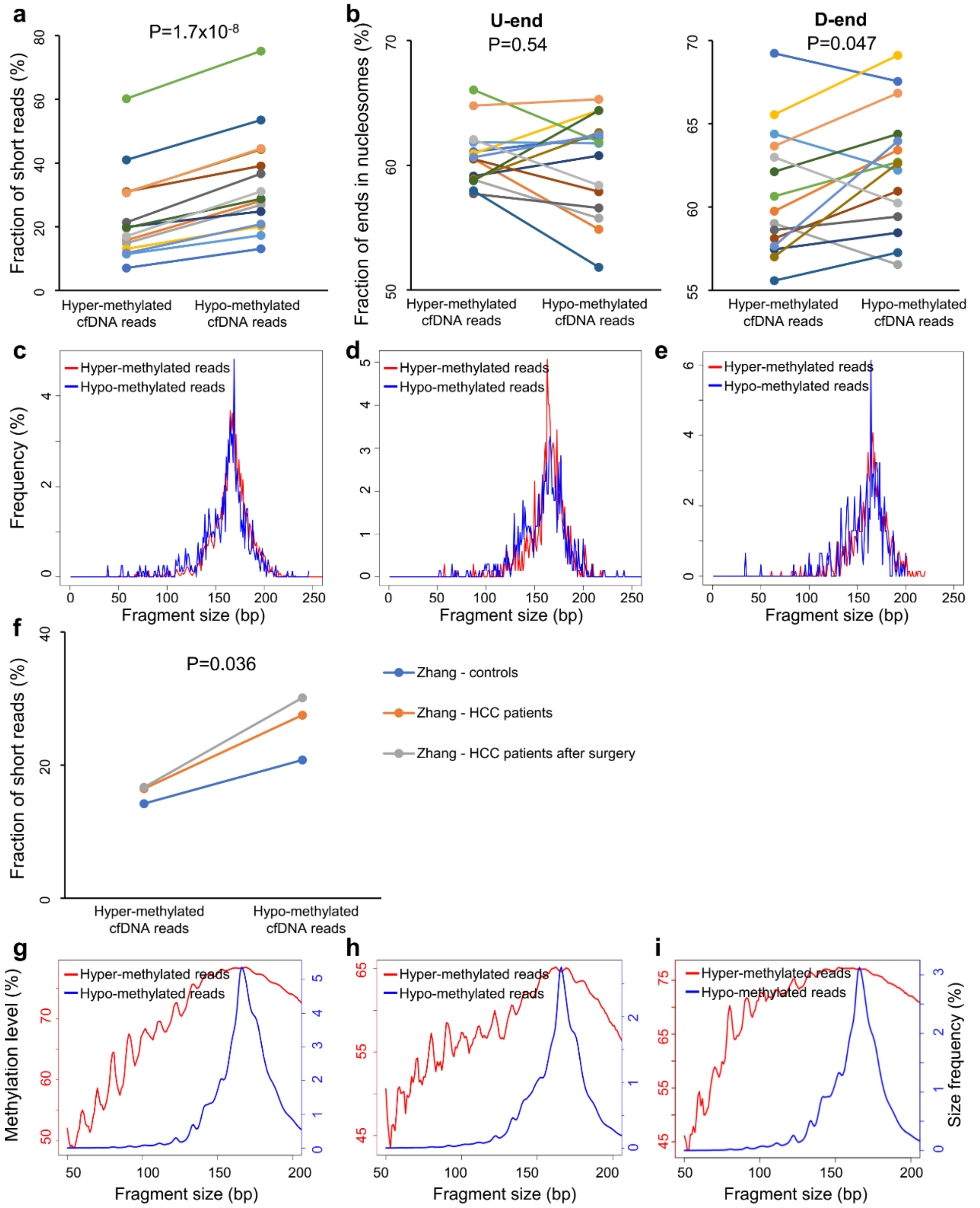

Supplementary Figure S5. Relationship between DNA methylation and cfDNA size.

(a) size distribution of hyper- and hypo-methylated cfDNA fragments (n=15 patients); (b) fraction of U- and D-ends within nucleosomes in hyper- and hypo-methylated cfDNA fragments (n=15 patients); (c) size distribution of hyper- and hypo-methylated cfDNA fragments in controls, HCC patients (d) before and (e) after surgery in Zhang *et*

al. dataset; (f) fraction of short reads in hyper- and hypo-methylated cfDNA fragments in Zhang *et al.* dataset (n=3 disease types); (g-i) genomewide distribution of cfDNA size and methylation level in (g) controls, HCC patients (h) before and (i) after surgery in Zhang *et al.* dataset. In (a-b), each dot represented 1 case; in (a, b, f), P-values were calculated using Mann-Whitney U tests (two-sided).

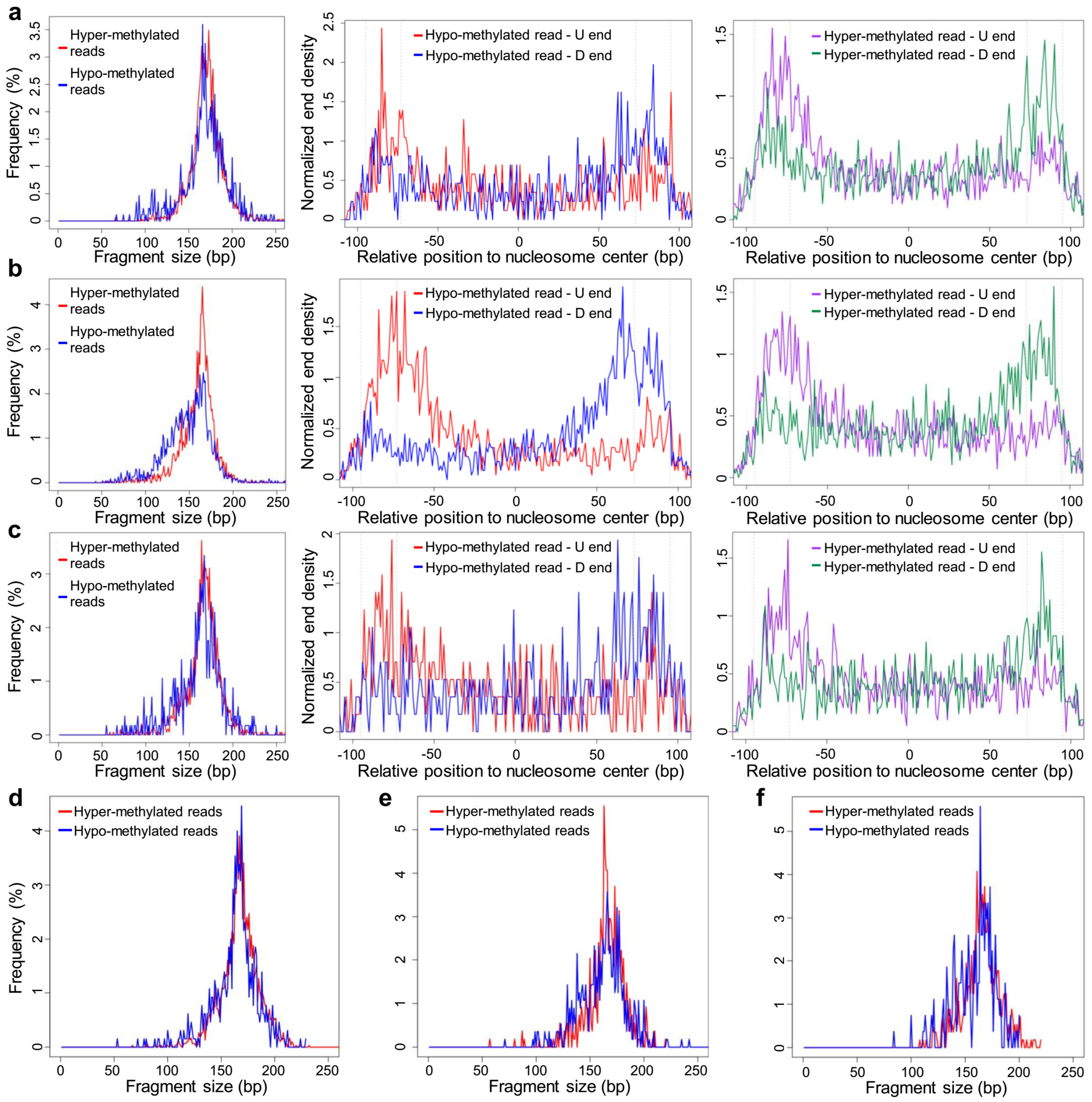

Supplementary Figure S6. Relationship between DNA methylation and cfDNA size using reads covering at least 5 CpGs. In (a) control subjects, (b) HCC patients, (c) lung cancer patients, the size distribution of hyper-and hypo-methylated cfDNA reads (left), and orientation-aware fragmentation end distribution for hypo- (middle) and hyper-methylated cfDNA in the nucleosomal context in chr12p11.1 loci; (d-f) size distribution of hypo- and hyper-methylated cfDNA fragments in (d) controls, (e) HCC patients (f) before and (i) after surgery in Zhang *et al.* dataset.

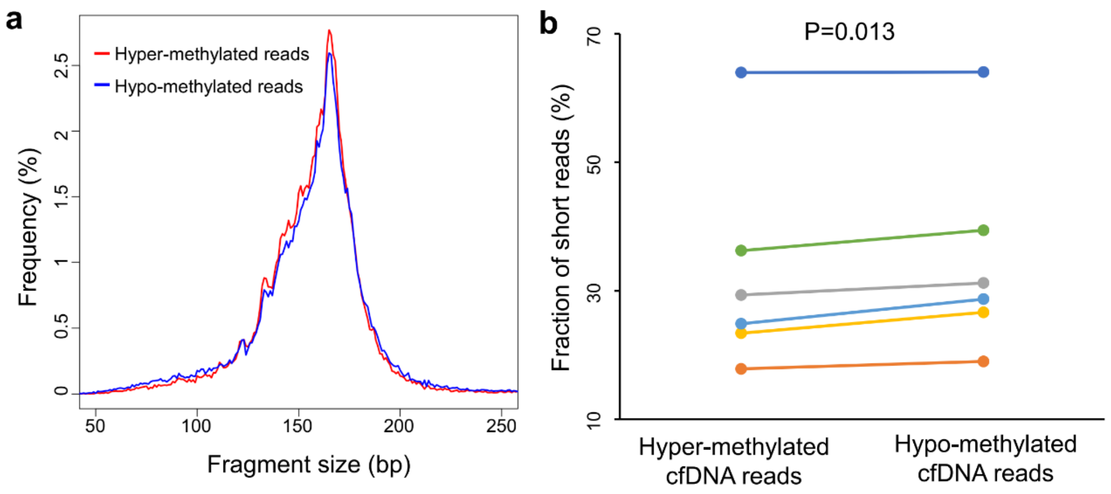

Supplementary Figure S7. Size pattern of cfDNA reads covering liver-specifically hyper-methylated CpG sites. (a) size distribution, and (b) fraction of short reads in hyper- and hypo-methylated cfDNA fragments (n=6 patients). P-value was calculated using paired t-test (two-sided).

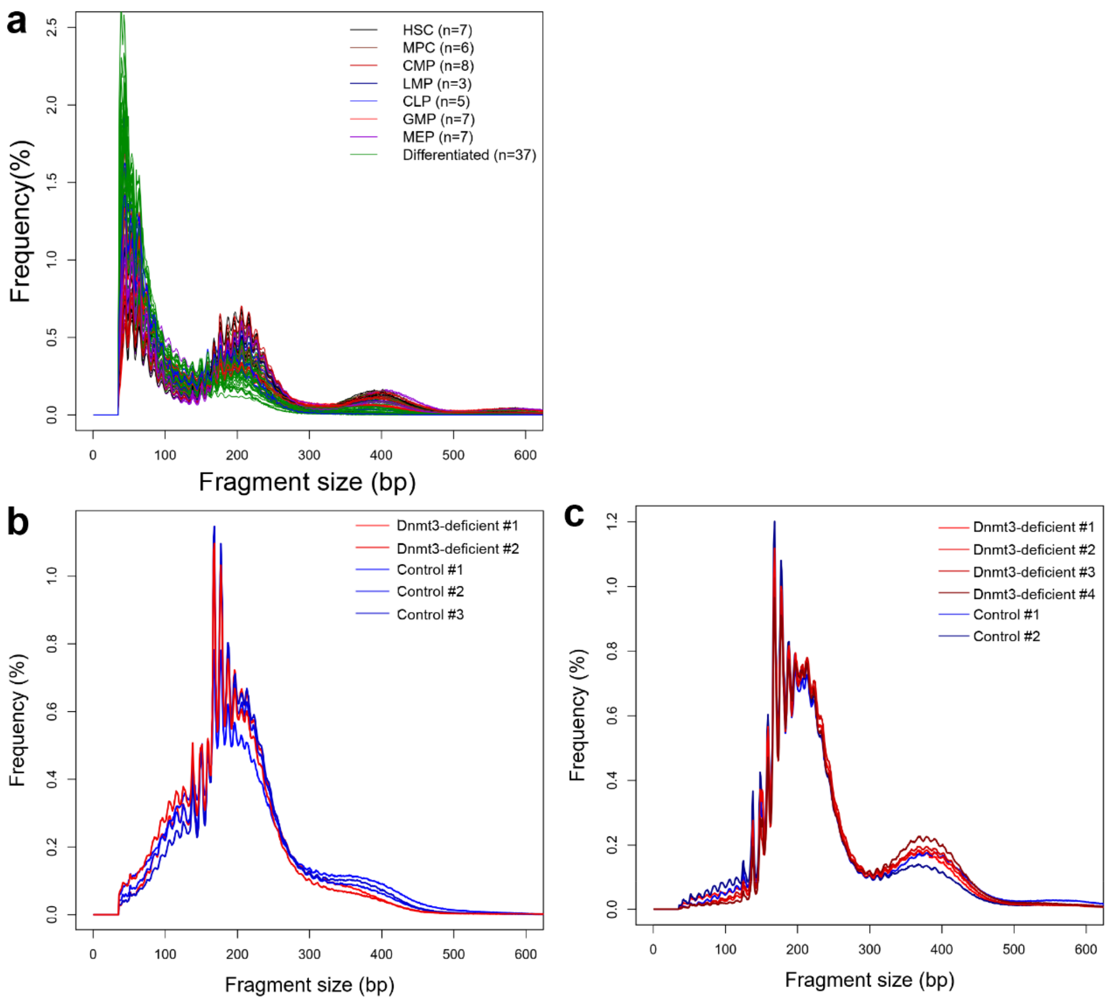

Supplementary Figure S8. Size distribution of Tn5-digested DNA fragments. (a) Corces *et al.* dataset working on hematopoiesis besides those shown in Fig. 4a, (b) naïve B-cell, and (c) germinal center B-cell in Barwick *et al.* dataset working on Dnmt3-deficient mouse model.

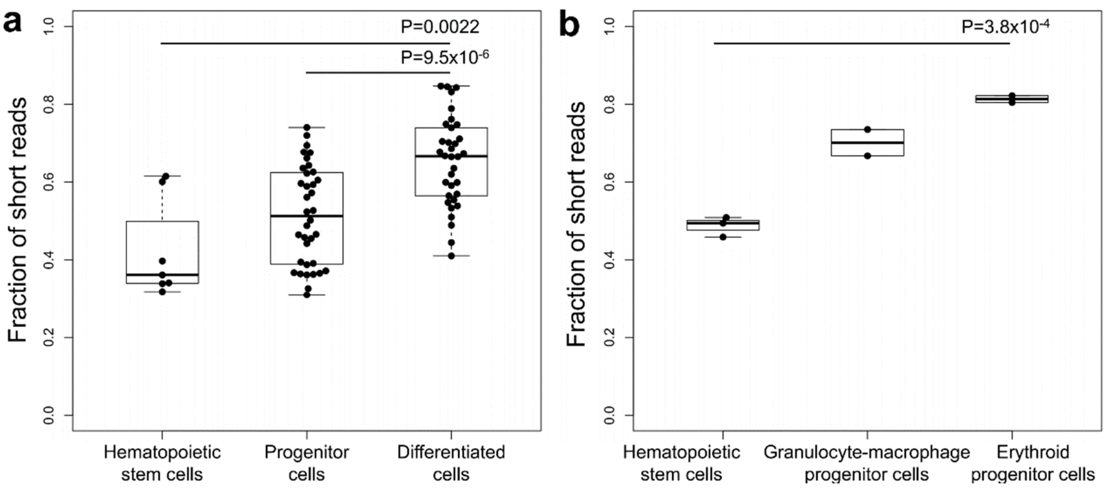

Supplementary Figure S9. Comparison of short read fractions among different cell types in (a) Corces *et al.* and (b) Vinny *et al.* datasets. Short reads were defined as reads shorter or equal to150 bp. P-values were calculated using t-tests (two-sided). Center line, median; box limits, 25th and 75th percentiles; whiskers, minimum to maximum.

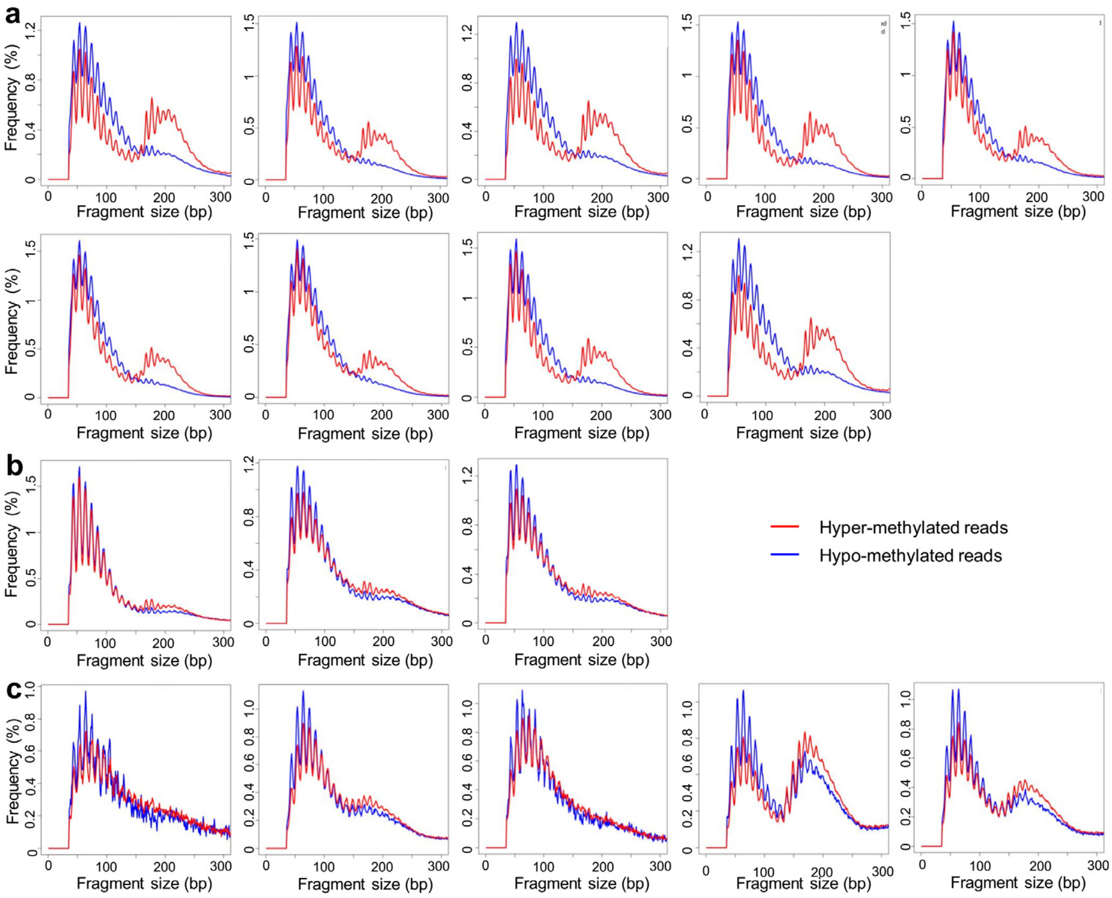

Supplementary Figure S10. Size distribution of Tn5-digested DNA fragments between different methylation levels in (a) Barnett *et al.*, (b) Lhoumaud *et al.*, (c) Izzo *et al.* datasets besides the ones shown in Fig. 4d-f.

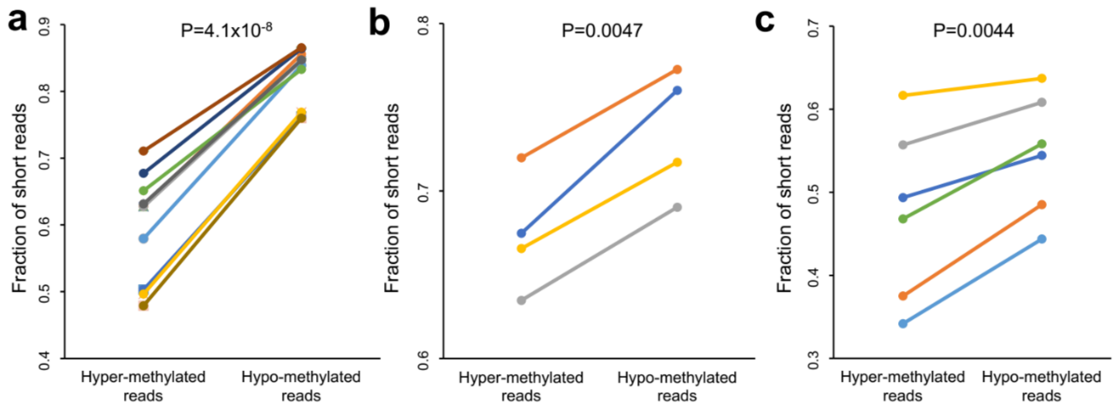

Supplementary Figure S11. Fraction of short reads between different DNA methylation levels in (a) Barnett *et al.* (n=10), (b) Lhoumaud *et al.* (n=4), (c) Izzo *et al.* (n=6) datasets. Short reads were defined as reads shorter or equal to 150 bp. P-values were calculated using paired t-tests (two-sided).

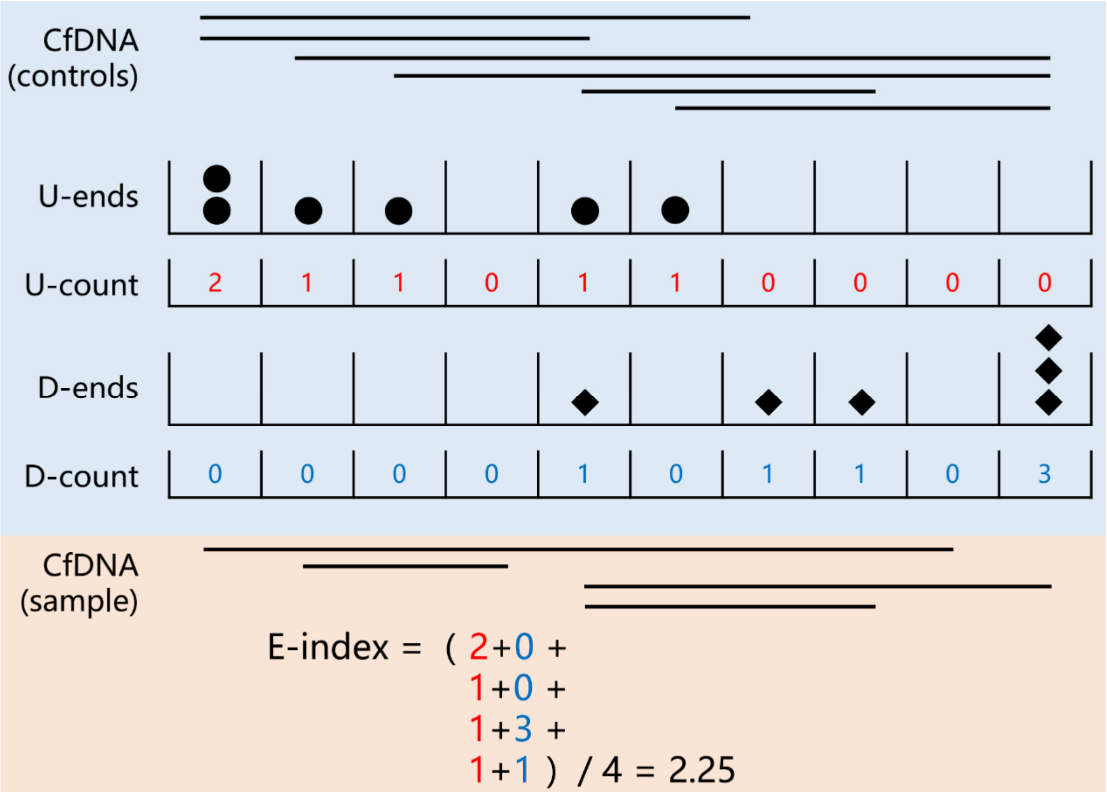

Supplementary Figure S12. Illustration of the E-index metric. Upper panel showed the establishment of orientation-aware end model using controls and the lower panel showed the calculation of E-index for a testing sample.

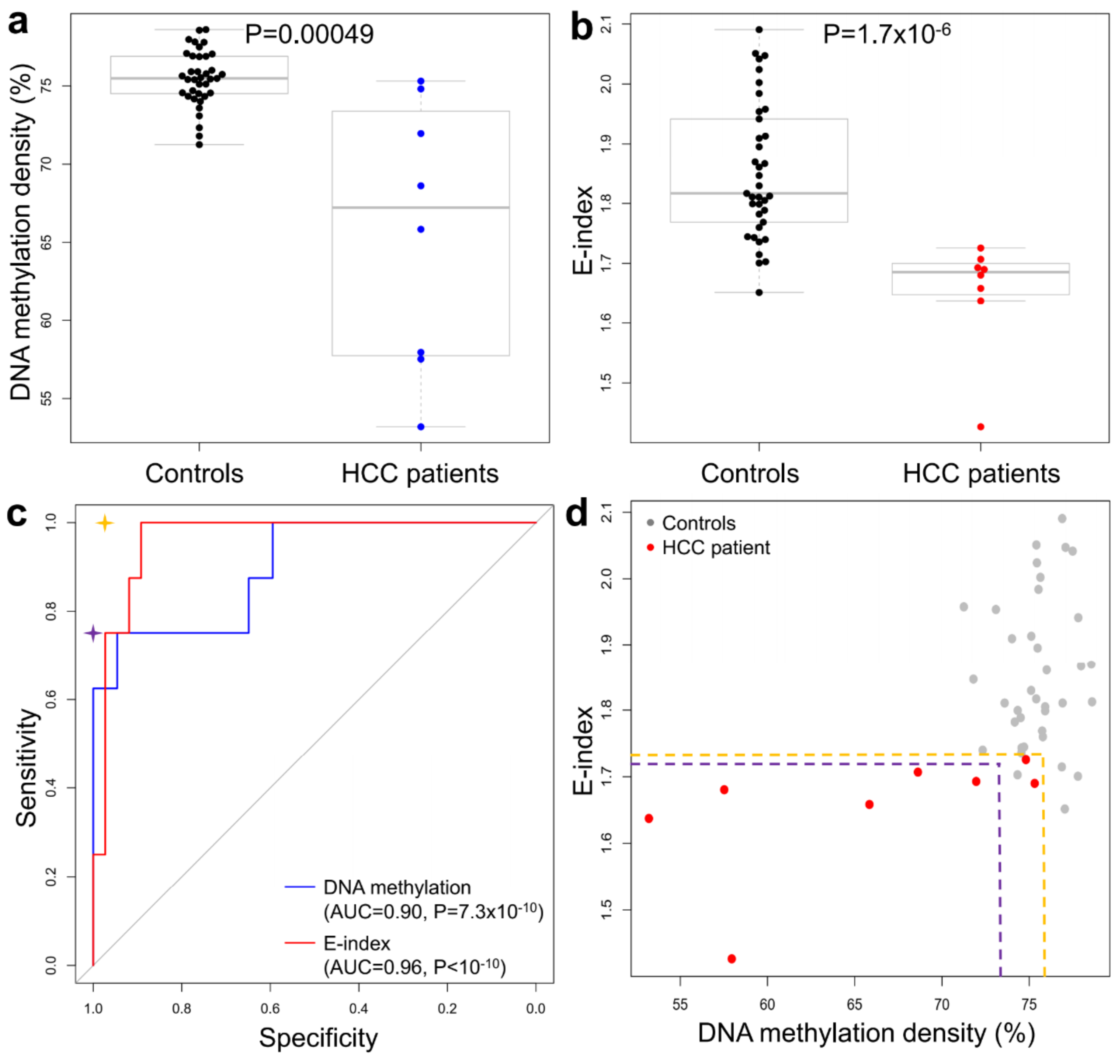

e

	Sensitivity at 100% specificity	Specificity at 100% sensitivity
DNA methylation	62.5 %	59.5 %
E-index	25.0 %	89.2 %
Combined	75.0 %	97.3 %

Supplementary Figure S13. E-index in Zhang *et al.* HCC dataset. (a) DNA methylation densities, and (b) E-index values in controls and HCC patients; (c) AUCs of DNA methylation and E-index in cancer diagnosis; (d) combination of DNA methylation and E-index, and diagnostic methods for cancer diagnosis; (e) performance of DNA methylation, E-index, and combination of these two in cancer diagnosis. Stars in (c) corresponded to diagnostic methods in (d): purple and yellow star/box represent an approach with 100% specificity and 100% sensitivity, respectively. In (a, b), P-

values were calculated using Mann-Whitney U tests; center line, median; box limits, 25th and 75th percentiles; whiskers, minimum to maximum; in (c), P-values were calculated using Z-tests; all P-vlaues are two-sided.
