# Supplementary material for: DNA methylation analysis explores the molecular basis of plasma cell-free DNA fragmentation
Source: Nat Commun. 2023 Jan 18;14:287. doi: 10.1038/s41467-023-35959-6 (PMC9849216; doi:10.1038/s41467-023-35959-6)
Supplement: Supplementary file 2 — Reporting Summary [file 41467_2023_35959_MOESM2_ESM.pdf]

nature portfolio

Corresponding author(s): Xin Jin, Kun Sun

Last updated by author(s): Nov 27, 2022

Reporting Summary

Nature Portfolio wishes to improve the reproducibility of the work that we publish. This form provides structure for consistency and transparency in reporting. For further information on Nature Portfolio policies, see our Editorial Policies and the Editorial Policy Checklist.

Statistics

For all statistical analyses, confirm that the following items are present in the figure legend, table legend, main text, or Methods section.

n/a	Confirmed	
☐	☒	The exact sample size (*n*) for each experimental group/condition, given as a discrete number and unit of measurement
☐	☒	A statement on whether measurements were taken from distinct samples or whether the same sample was measured repeatedly
☐	☒	The statistical test(s) used AND whether they are one- or two-sided<br>*Only common tests should be described solely by name; describe more complex techniques in the Methods section.*
☒	☐	A description of all covariates tested
☒	☐	A description of any assumptions or corrections, such as tests of normality and adjustment for multiple comparisons
☐	☒	A full description of the statistical parameters including central tendency (e.g. means) or other basic estimates (e.g. regression coefficient) AND variation (e.g. standard deviation) or associated estimates of uncertainty (e.g. confidence intervals)
☐	☒	For null hypothesis testing, the test statistic (e.g. *F*, *t*, *r*) with confidence intervals, effect sizes, degrees of freedom and *P* value noted<br>*Give P values as exact values whenever suitable.*
☒	☐	For Bayesian analysis, information on the choice of priors and Markov chain Monte Carlo settings
☒	☐	For hierarchical and complex designs, identification of the appropriate level for tests and full reporting of outcomes
☐	☒	Estimates of effect sizes (e.g. Cohen's *d*, Pearson's *r*), indicating how they were calculated

Our web collection on statistics for biologists contains articles on many of the points above.

Software and code

Policy information about availability of computer code

Data collection	No software was used. Statistical analysis was performed using R software (v3.6.2).
Data analysis	CfDNA whole genome sequencing data, ATAC-seq data and cfMeDIP-seq data were analyzed using a unified pipeline: the raw reads were firstly preprocessed using Ktrim software (https://github.com/hellosunking/Ktrim, v1.3.0) to remove sequencing adapter and low-quality cycles; the preprocessed reads were then mapped to reference human genome (NCBI GRCh38) for human samples, reference mouse genome (NCBI GRCm38) for normal mouse samples, or a pseudo-genome that combined reference human and mouse genomes for PDX samples, using Bowtie2 software (https://bowtie-bio.sourceforge.net/bowtie2, v2.3.5.1); PCR duplications (i.e., reads with identical ending positions) were identified and removed using in-house programs, and resulting reads were collected as the final clean data. Due to the limited depth for each case, in each dataset, cfDNA samples from the same cancer type or the control group were pooled together during downstream fragmentation analyses. For PDX samples, reads mapped to human genome were considered as tumor-derived and were used in the downstream analyses. For Liang et al. dataset, tumor DNA load in plasma cfDNA was estimated using ichorCNA software (https://github.com/broadinstitute/ichorCNA, v0.2.0).<br><br>EM-seq, WGBS, and Tn5-digestion followed by bisulfite-sequencing datasets were analyzed using Msuite2 software (https://github.com/hellosunking/Msuite2, v2.1.0), which included quality control, read alignment, and methylation call. For EM-seq and WGBS datasets, sequencing reads covering at least 2 CpG sites with an average methylation level larger than 80% or lower than 20% were considered as hypermethylated or hypomethylated reads, respectively. For ATAC-seq and Tn5-digestion followed by bisulfite-sequencing datasets, as we were only interested in Tn5 cutting within nucleosomes, only reads outside the peak regions (i.e., open-chromatin regions that do not have nucleosomes; obtained from the corresponding studies) were used in downstream analyses.<br><br>Nucleosome track for GM12878 cell line (lymphoblastoid lineage) was downloaded from NucMap database (https://ngdc.cncb.ac.cn/nucmap;

accession number: hsNuc0390101; nucleosome occupancy and center loci were determined using DANPOS software (https://github.com/sklasfeld/DANPOS3)).

Computational programs and scripts to reproduce the results were available at https://github.com/hellosunking/molecular-cfDNA-fragmentomics .

For manuscripts utilizing custom algorithms or software that are central to the research but not yet described in published literature, software must be made available to editors and reviewers. We strongly encourage code deposition in a community repository (e.g. GitHub). See the Nature Portfolio guidelines for submitting code & software for further information.

Data

Policy information about availability of data

All manuscripts must include a data availability statement. This statement should provide the following information, where applicable:

- Accession codes, unique identifiers, or web links for publicly available datasets
- A description of any restrictions on data availability
- For clinical datasets or third party data, please ensure that the statement adheres to our policy

Raw sequencing data reported in this paper had been deposited in the Genome Sequence Archive in National Genomics Data Center, China National Center for Bioinformation / Beijing Institute of Genomics, Chinese Academy of Sciences (GSA-Human: HRA002237, HRA002250, and HRA002298) that are publicly accessible at https://ngdc.cncb.ac.cn/gsa-human.

CfDNA whole genome sequencing datasets were downloaded from Gene Expression Omnibus (GEO; accession numbers: GSE71378, GSE124686, and GSE81314; note that we only analyzed the data generated using double-strand cfDNA) and FinaleDB72; cfDNA WGBS dataset was downloaded from Genome Sequence Archive in National Genomics Data Center (GSA; accession number: CRA001537); ATAC-seq datasets were downloaded from GEO (accession numbers: GSE74912, GSE138003, and GSE89471); Tn5-digestion followed by bisulfite-sequencing datasets were downloaded from GEO (accession numbers: GSE130096, GSE124822, and GSE129673); cfMeDIP-seq datasets were downloaded from GEO (accession numbers: GSE79838, GSE152631; all sequenced in single-end mode) and Sequence Read Archive (SRA; accession number: SRP262262; all sequenced in single-end mode).

Reference human genome (NCBI GRCh38) and mouse genome (NCBI GRCm38) was obtained from UCSC genome browser (https://hgdownload.cse.ucsc.edu/goldenpath/hg38/bigZips/hg38.fa.gz and https://hgdownload.cse.ucsc.edu/goldenpath/mm10/bigZips/mm10.fa.gz)

Human research participants

Policy information about studies involving human research participants and Sex and Gender in Research.

Reporting on sex and gender	Not collected. Sex information is irrelevant to this work.
Population characteristics	Non-cancer controls (age: 30-56, median: 42), Hepatocellular carcinoma patients (age: 48-78, median: 61), Lung adenocarcinoma patients (age: 40-68, median: 65).
Recruitment	Participants were recruited from The Third People's Hospital of Shenzhen and Peking Union Medical College Hospital. Participants were randomly recruited without selection.
Ethics oversight	This study had been approved by the Ethics Committee of Shenzhen Bay Laboratory and Ethics Committee of The Third People's Hospital of Shenzhen.

Note that full information on the approval of the study protocol must also be provided in the manuscript.

Field-specific reporting

Please select the one below that is the best fit for your research. If you are not sure, read the appropriate sections before making your selection.

[x] Life sciences [ ] Behavioural & social sciences [ ] Ecological, evolutionary & environmental sciences

For a reference copy of the document with all sections, see nature.com/documents/nr-reporting-summary-flat.pdf

Life sciences study design

All studies must disclose on these points even when the disclosure is negative.

Sample size	No sample size calculation was performed. This is an observational study.
Data exclusions	1 sample in Xu et al. dataset was discarded due to aberrant size pattern.
Replication	All findings were replicated using at least 2 datasets from different groups.
Randomization	Not relevant to this study. Participates of the same type (e.g., control, hepatocellular carcinoma) were used as 1 category without sub-

	grouping.
Blinding	The investigators were not blinded to data since we need to known the clinical diagnosis of each patient to group the samples and perform comparisons between each group.

Reporting for specific materials, systems and methods

We require information from authors about some types of materials, experimental systems and methods used in many studies. Here, indicate whether each material, system or method listed is relevant to your study. If you are not sure if a list item applies to your research, read the appropriate section before selecting a response.

Materials & experimental systems

n/a	Involved in the study	
☒	☐	Antibodies
☒	☐	Eukaryotic cell lines
☒	☐	Palaeontology and archaeology
☐	☒	Animals and other organisms
☒	☐	Clinical data
☒	☐	Dual use research of concern

Methods

n/a	Involved in the study	
☒	☐	ChIP-seq
☒	☐	Flow cytometry
☒	☐	MRI-based neuroimaging

Animals and other research organisms

Policy information about studies involving animals; ARRIVE guidelines recommended for reporting animal research, and Sex and Gender in Research

Laboratory animals	Immunocompromised NOD/SCID gamma (NSG) mice (8 weeks old) were used. Mice were housed under specific pathogen-free conditions with a 12h light/dark cycle, at a temperature of 20-26°C, and a relative humidity of 40-70%; mice were fed a standard mouse chow diet.
Wild animals	The study did not involve wild animals.
Reporting on sex	Not collected in this study.
Field-collected samples	Blood.
Ethics oversight	Animal study was conducted according to protocols approved by the Institutional Animal Care and Use Committee, Southern University of Science and Technology.

Note that full information on the approval of the study protocol must also be provided in the manuscript.
